# Comprehensive microRNA-sequencing of exosomes derived from head and neck carcinoma cells *in vitro* reveals common secretion profiles and potential utility as salivary biomarkers

**DOI:** 10.18632/oncotarget.19614

**Published:** 2017-07-27

**Authors:** Scott Langevin, Damaris Kuhnell, Tess Parry, Jacek Biesiada, Shouxiong Huang, Trisha Wise-Draper, Keith Casper, Xiang Zhang, Mario Medvedovic, Susan Kasper

**Affiliations:** ^1^ Department of Environmental Health, University of Cincinnati College of Medicine, Cincinnati, OH, USA; ^2^ Physical and Computational Sciences Department, Bethany College, Bethany, WV, USA; ^3^ Division of Hematology/Oncology, Department of Internal Medicine, University of Cincinnati College of Medicine, Cincinnati, OH, USA; ^4^ Department of Otolaryngology, University of Michigan, Ann Arbor, MI, USA

**Keywords:** HNSCC, microvesicles, extracellular vesicles, miRNA, liquid biopsy

## Abstract

Exosomes are nano-scale, membrane encapsulated vesicles that are released by cells into the extracellular space and function as intercellular signaling vectors through horizontal transfer of biologic molecules, including microRNA (miRNA). There is evidence that cancer-derived exosomes enable the tumor to manipulate its microenvironment, thus contributing to the capacity of the tumor for immune evasion, growth, invasion, and metastatic spread. The objective of this study was to characterize differential secretion of exosomal miRNA by head and neck squamous cell carcinoma (HNSCC) and identify a set of candidate biomarkers that could be detected in non-invasive saliva samples. We isolated exosomes from conditioned media from 4 HNSCC cell lines and oral epithelial control cells and applied miRNA-sequencing to comprehensively characterize their miRNA cargo and compare transcript levels of each HNSCC cell line to that of oral epithelial control cells. A candidate set of miRNA differentially secreted by all 4 HNSCC cell lines was further evaluated in saliva collected from HNSCC patients and healthy controls. We observed extensive differences in exosomal miRNA content between HNSCC cells when compared to normal oral epithelial control cells, with a high degree of overlap in exosomal miRNA profiles between the 4 distinct HNSCC cell lines. Importantly, several of the exosomal miRNA secreted solely by cancer cells in culture were detected at substantially elevated levels in saliva from HNSCC patients relative to saliva from healthy controls. These findings provide important insight into tumor biology and yields a promising set of candidate HNSCC biomarkers for use with non-invasive saliva samples.

## INTRODUCTION

Head and neck cancer is the 10^th^ most common malignancy overall and 5^th^ most common among men in the United States [1], of which more than 90% are histologically squamous (HNSCC) [2]. About two-thirds of HNSCC patients are diagnosed at an advanced stage [3], and more than half suffer at least one recurrence, with∼90% of those occurring within 2-years of initial treatment [4–6]. Understandably, there is considerable interest in discerning the impetus(es) for the genesis of both primary and recurrent tumors, as well as discovery and development of novel biomarkers to facilitate earlier detection.

Exosomes are nano-scale, membrane encapsulated vesicles of about 40-150 nm in diameter [7, 8] that are released by cells into the extracellular space and function as intercellular signaling vectors through horizontal transfer of biomolecules [9]. Included among this biomolecular cargo are microRNA (miRNA) [10], which are small, evolutionarily conserved, non-coding RNA (ncRNA) ranging from 18-25 nucleotides in length. These represent an important class of ncRNA, as they are involved in negative regulation of gene expression in essentially all eukaryotic organisms through post-transcriptional degradation and translational inhibition of messenger RNA (mRNA), with estimates suggesting that up to 60% of human protein coding genes are regulated by miRNA [11]. Exosomes are secreted by both normal and malignant cells [8], and the biomolecular cargo is dependent upon the cell of origin [9]. Since they are secreted into the intercellular space, exosomes can be detected in a variety of extracellular biologic fluids, including serum/plasma, urine, and saliva. Importantly, there is emerging evidence that cancer-derived exosomes enable the tumor to manipulate its microenvironment, potentially contributing to its capacity for immune evasion, growth, invasion, and metastatic spread [12, 13], thus making them particularly attractive biomarker sources.

The purpose of this study was to employ comprehensive Next-Generation microRNA-sequencing (miRNA-seq) to catalog differentially secreted exosomal miRNA from HNSCC cells relative to non-pathologic oral epithelial cells. Specifically, we sought to (1) uncover common patterns in exosomal secretion of miRNA by HNSCC; and (2) identify a set of candidate biomarkers that can potentially be applied to saliva for early detection of primary or recurrent HNSCC.

## RESULTS

We cultured 4 discrete HNSCC cell lines (Table 1) that originated from 4 different sites in the upper aerodigestive tract: [1] H413 (buccal) [14]; [2] Detroit 562 (pharynx metastatic to pleura) [15]; [3] FaDu (hypopharynx) [16]; and [4] Cal 27 (tongue) [17]. We also cultured primary human gingival epithelial cells (HGEPp) that were pooled from 3 healthy female donors as a control for comparison. Briefly, cells were cultured in triplicate using exosome-depleted fetal bovine serum (FBS) when applicable. After reaching 80-90% confluence, media was harvested, and exosomes were isolated and purified via differential ultracentrifugation, using the protocol described by Gallo et al. [18].

**Table 1:** Description of the cells cultured during the *in vitro* aspect of this study

	H413	Detroit 562	FaDu	Cal 27	Normal oral epithelial cells
Organism	Human	Human	Human	Human	Human
Culture type	Cancer cell line	Cancer cell line	Cancer cell line	Cancer cell line	Primary, pooled^a^
Histopathology	Squamous cell carcinoma	Squamous cell carcinoma	Squamous cell carcinoma	Squamous cell carcinoma	Non-pathologic epithelium
Site of Origin	Buccal mucosa	Pharynx^b^	Hypopharynx	Tongue	Gingival mucosa
Age (years)	53	[unknown]	56	56	21, 21, 27
Sex	Female	Female	Male	Male	Female
Race/ethnicity	[unknown]	Caucasian	Caucasian	Caucasian	Caucasian

^a^Pooled sample of cells from 3 healthy female donors.

^b^Established from a pleural metastasis of pharyngeal carcinoma cells.

### Isolation and characterization of exosomes from conditioned culture media

**Figure 1:**
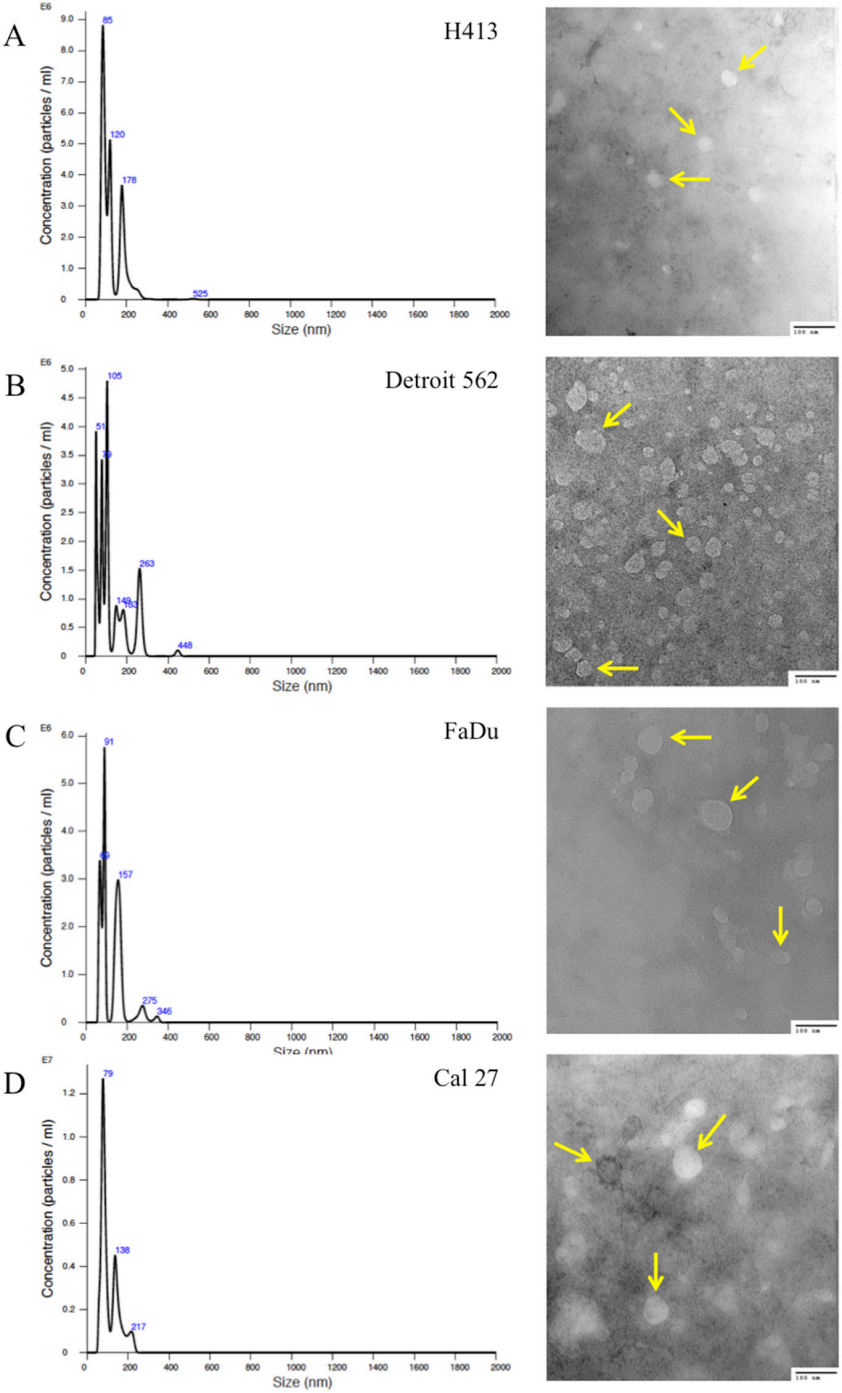
Size distribution plots from nanoparticle tracking analysis of exosome isolates from culture media of head and neck squamous cell carcinoma (HNSCC) cell lines **(A)** H413, **(B)** Detroit 562, **(C)** FaDu, and **(D)** Cal 27, according to particle diameter. Representative transmission electron microscopy (TEM; 200,000x) images of exosomes isolated from each respective HNSCC line are presented to the right of each plot; arrows highlight representative exosomes. A 100-nm scale bar is provided in the bottom right hand corner of each image for perspective.

**Figure 2:**
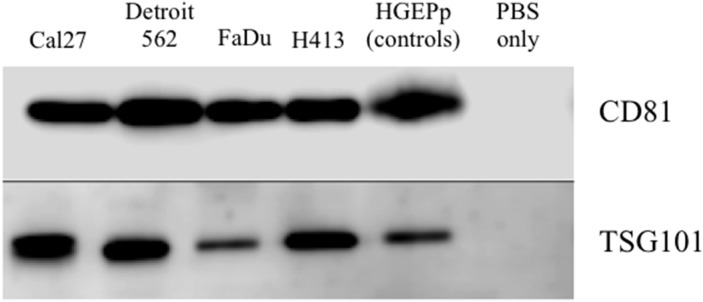
Western blot analysis of protein expression of exosome-associated tetraspanin CD81 and cytosolic endosomal sorting complex component TSG101 for exosome isolates from conditioned cell culture media for head and neck squamous carcinoma cell lines and primary non-pathologic oral epithelial cells No template controls (phosphate buffered saline (PBS) only) were included on each gel.

Exosomes were isolated from conditioned culture media for each of the HNSCC cell lines, as well as the normal primary oral epithelial cells. The presence of purified exosomes was visually confirmed by transmission electron microscopy (TEM), quantified via nanoparticle tracking analysis (NTA; Figure 1) and further verified by Western blot [19] (Figure 2). The NTA distribution plots illustrate the relative purity of these isolates, with the bulk of particles having a diameter < 200 nm. Although the distribution plots for Detroit 562 show a moderate peak at 263 nm in diameter, indicating some impurity, the majority of isolated particles (> 80%) were < 200 nm in diameter.

### Differential secretion of exosomal miRNA by HNSCC cells

**Figure 3:**
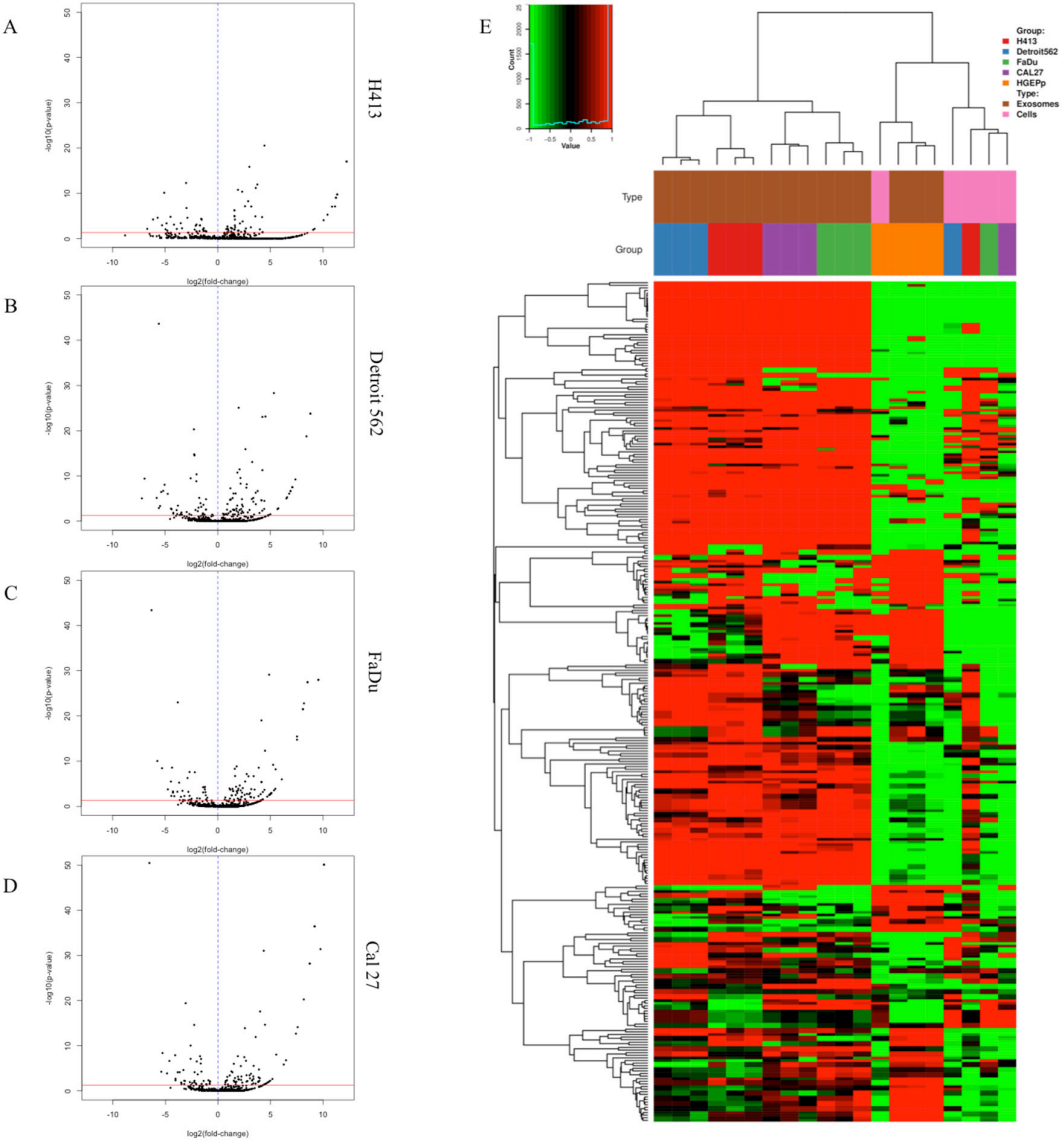
Secretion patterns of exosomal miRNA for head and neck squamous cell carcinoma (HNSCC) cell lines and primary non-pathologic oral epithelial control cells Volcano plots for differentially secreted miRNA in exosomes isolated from conditioned cell culture media are depicted for (**A**) H413, (**B**) Detroit 562, (**C**) FaDu, and (**D**) Cal 27 head and HNSCC cells relative to non-pathologic oral epithelial control cells. The horizontal red line in each plot corresponds to p = 0.05, adjusted for false discovery rate (FDR). Each black dot represents a specific miRNA transcript; those to the right of the vertical blue dashed line correspond to a relative increase in secreted level by the HNSCC cells and those to the left correspond to a relative decrease in secreted level. (**E**) Heatmap of miRNA profile of exosomes (each in triplicate) or intracellular expression for each HNSCC cell line or non-pathologic oral epithelial control cells. Each column represents a different sample and rows represent miRNA transcripts. Source (exosomal or intracellular) and cell type correspond to the key at the top right of the figure.

We performed miRNA-seq on total RNA extracted from exosome isolates to comprehensively characterize the exosomal miRNA secretome of HNSCC and non-pathologic oral epithelial cells and identify differential secretion profiles common to HNSCC. Extensive differences in exosomal miRNA content were observed between each respective HNSCC cell line and oral epithelial control cells, as shown by the volcano plots presented in Figure 3A–3D. A total of 134 mature miRNA were differentially secreted in exosomes by one or more HNSCC cell lines relative to the oral epithelial control cells (Q ≤ 0.1). A number of commonalities in exosomal secretion profiles of miRNA were observed across the 4 HNSCC cell lines and were in stark contrast with those of the oral epithelial control cells. This is highlighted in the heatmap in Figure 3E, which depicts exosomal (in triplicate) and intracellular miRNA (pooled) profiles from each HNSCC cell line and primary oral epithelial cells and shows the exosomal secretion profile of miRNA profile from each cell line (and respective replicates) all clustering together. Of particular note are the large blocks of exosomal miRNA for which secretion is differentially upregulated across the HNSCC cell lines relative to the control cells. Additionally, respective exosomal and intracellular miRNA profiles within each cell type are clearly distinct, further supporting the notion that the loading of exosomal cargo is an active, highly regulated process rather than a passive process that merely reflects cellular content [20].

**Figure 4:**
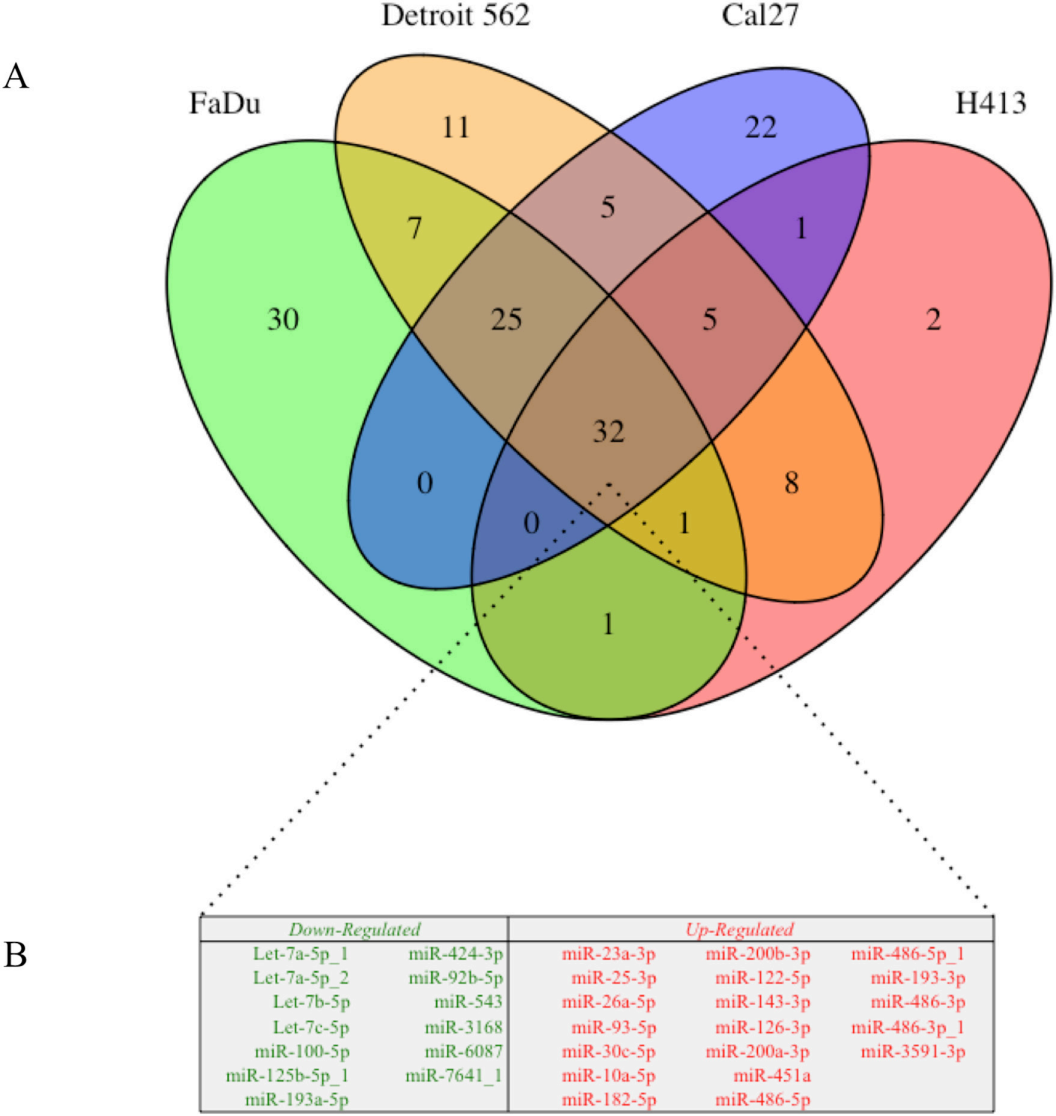
**(A)** Venn diagram of differential exosomal miRNA cargo for each of 4 head and neck squamous cell carcinoma (HNSCC) cell lines relative to those derived from primary non-pathologic oral epithelial control cells; numbers in each segment represent the respective number of overlapping differentially secreted exosomal miRNA. **(B)** Exosomal miRNA that were differentially secreted in exosomes by all 4 HNSCC cell lines (n = 32) relative to the oral epithelial control cells. Downregulated exosomal miRNA (n = 13) are presented in green and upregulated miRNA (n = 19) are presented in red.

The overlap of differentially secreted exosomal miRNA (relative to the oral epithelial cells) between HNSCC cell lines is illustrated in Figure 4. There were 101 transcripts that were differentially secreted by ≥ 2 of the HNSCC lines (69 upregulated, 32 downregulated), 63 differentially secreted by ≥ 3 of the HNSCC lines (40 upregulated, 23 downregulated), and 32 differentially secreted by all 4 HNSCC cell lines (19 upregulated, 13 downregulated). Somewhat surprisingly there was complete agreement with regard to the direction of effect for overlapping differentially secreted miRNA among the respective HNSCC cell lines.

Of particular interest from the perspective of biomarker discovery was the observation that 22 miRNA transcripts were detected in exosomes from at least 1 HNSCC cell line but were not secreted by the oral epithelial control cells (Supplementary Table 1). More notably, 2 such transcripts were secreted in exosomes from 3 out of 4 HNSCC lines (*miR-223-3p* and *miR-150-5p*), and 8 were secreted by all 4 HNSCC lines (*miR-122-5p*, *miR-143-3p*, *miR-451a*, *miR-486-5p*, *miR-486-5p_1*, *miR-486-3p*, *miR-486-3p_1*, and *miR-3591-3p*).

### Functional miRNA pathway analysis

To assess the potential role of exosomal miRNA in the context of HNSCC tumor biology and microenvironment, pathway enrichment analysis was conducted using DIANA mirPath v.3 [21] for the common subset of exosomal miRNA that were differentially secreted by all HNSCC cell lines (n = 32). Analyses were performed for Kyoto Encyclopedia of Genes and Genomes (KEGG) pathways and Gene Ontology (GO) biological processes based on experimentally validated [22] and computationally predicted [23] gene targets. The subset of miRNA differentially secreted by all 4 HNSCC cell lines was enriched for those targeting genes involved in major cancer-associated KEGG pathways, including cell signaling (notably: p53-, TGF-ß, Hippo, Ras, MAPK, PI3K-Akt, and Ergß signaling pathways) and key cellular functions, including cell cycle control, RNA splicing, and cellular adhesion (Supplementary Tables 2-5). The KEGG pathway “*Proteoglycans in cancer*” was amongst the top hits irrespective of whether predicted (p_enrichment_ = 2.78×10^-10^) or validated (p_enrichment_ = 1.18×10^-14^) miRNA targets were used. There was a heavy enrichment of GO biological processes related to metabolism/catabolism/biosynthesis and immunity, in particular those involved with immune stimulation, such as toll-like and Fc receptors (Table 2). Also of note was the enrichment of miRNA targeting genes involved in signal transduction (e.g. EGFR and FGFR receptor tyrosine kinase signaling pathways), apoptosis, cell cycle, transcription, cell motility, extracellular matrix disassembly/organization processes, and RNA splicing (Supplementary Tables 2-5).

**Table 2:** Enrichment for biological processes related to *metabolism, catabolism & synthesis* and *immune function* among functionally validated targets of miRNA that were differentially secreted via exosomes across all 4 HNSCC lines relative to those from non-pathologic oral epithelial cells

GO biological process	Enrichment p-value
*Metabolism, Catabolism & Biomolecular Synthesis*	
Cellular nitrogen compound metabolic process	4.51E-132
Biosynthetic process	2.98E-100
Small molecule metabolic process	6.52E-33
Catabolic process	1.71E-24
Cellular lipid metabolic process	6.40E-18
Cellular protein metabolic process	3.16E-16
Glycosaminoglycan metabolic process	2.98E-11
Nucleobase-containing compound catabolic process	2.42E-10
Energy reserve metabolic process	1.34E-09
Chondroitin sulfate metabolic process	1.78E-05
Sulfur compound metabolic process	9.17E-05
mRNA metabolic process	1.05E-04
Hexose transport	4.99E-04
Vitamin metabolic process	5.19E-04
Generation of precursor metabolites and energy	7.34E-04
Water-soluble vitamin metabolic process	1.12E-03
DNA metabolic process	1.17E-03
Phospholipid metabolic process	1.56E-03
Glycerophospholipid biosynthetic process	2.22E-03
Nucleobase-containing small molecule metabolic process	2.42E-03
Regulation of glucose transport	2.95E-03
RNA metabolic process	2.95E-03
Glutamate secretion	4.09E-03
Regulation of insulin secretion	5.07E-03
Nuclear-transcribed mRNA catabolic process, deadenylation-dependent decay	5.94E-03
Phosphatidylinositol biosynthetic process	8.03E-03
Inositol phosphate metabolic process	1.04E-02
Dolichol-linked oligosaccharide biosynthetic process	1.50E-02
Unsaturated fatty acid metabolic process	3.08E-02
Alpha-linolenic acid metabolic process	3.08E-02
Keratan sulfate metabolic process	4.32E-02
Regulation of cellular amino acid metabolic process	4.69E-02
*Immune Function*	
Fc-epsilon receptor signaling pathway	1.42E-37
TRIF-dependent toll-like receptor signaling pathway	5.92E-27
Toll-like receptor 10 signaling pathway	1.08E-22
MyD88-independent toll-like receptor signaling pathway	1.25E-22
Toll-like receptor TLR1:TLR2 signaling pathway	1.46E-22
Toll-like receptor TLR6:TLR2 signaling pathway	1.46E-22
Fc-gamma receptor signaling pathway involved in phagocytosis	2.37E-19
Toll-like receptor 3 signaling pathway	2.91E-19
Toll-like receptor 5 signaling pathway	1.40E-18
Immune system process	4.31E-18
Toll-like receptor 9 signaling pathway	1.07E-17
Platelet activation	1.34E-17
Toll-like receptor 4 signaling pathway	3.60E-15
Toll-like receptor 2 signaling pathway	3.85E-14
Toll-like receptor signaling pathway	2.50E-13
MyD88-dependent toll-like receptor signaling pathway	4.31E-09
Platelet degranulation	8.94E-09
Leukocyte migration	1.02E-08
Innate immune response	1.59E-07
Antigen processing and presentation of exogenous peptide antigen via MHC class I	2.46E-07
Antigen processing and presentation of exogenous peptide antigen via MHC class I, TAP-dependent	3.80E-07
Antigen processing and presentation of exogenous peptide antigen via MHC class II	2.19E-06
Positive regulation of type I interferon production	1.49E-05
Negative regulation of type I interferon production	1.56E-03
Cytokine-mediated signaling pathway	3.24E-03
Antigen processing and presentation of peptide antigen via MHC class I	1.25E-02
Regulation of interferon-gamma-mediated signaling pathway	1.90E-02

### Translation to human saliva samples

#### miRNA-sequencing pilot

**Figure 5:**
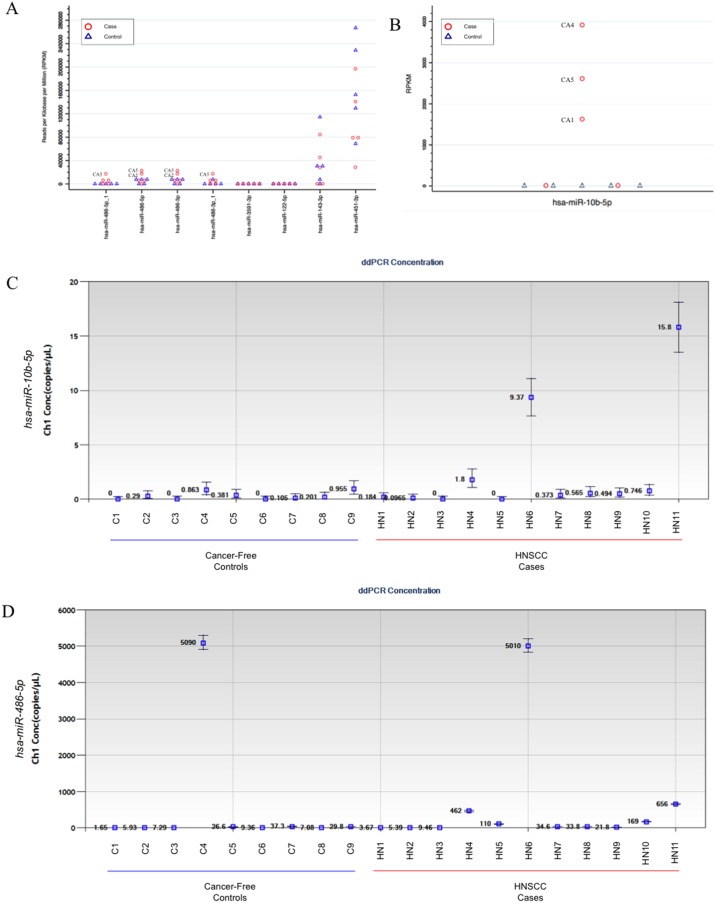
Secretion levels of candidate exosomal miRNA identified in the *in vitro* work in saliva from head and neck squamous cell carcinoma (HNSCC) patients and healthy controls The miRNA-sequencing data used to guide selection of ddPCR assay development based on reads per kilobase per million transcripts (RPKM) for exosomal **(A)** *miR-486-5p*, *miR-486-5p_1*, *miR-486-3p*, *miR-486-3p_1*, *miR-122-5p*, *miR-143-3p*, *miR-451a*, and *miR-3591-3p*, and **(B)** *miR-10b-5p* transcripts isolated from saliva samples obtained from a miRNA-sequencing data from a pilot study involving 5 HNSCC cases and 5 cancer-free controls. ddPCR results from TaqMan assays for **(C)** *miR-10b-5p* and **(D)** *miR-486-5p* performed on salivary exosomes isolates from HNSCC cases (n = 11) and controls (n = 9).

As a preliminarily assessment of the potential for clinical utility of these candidate markers in non-invasive saliva samples and to guide the selection of miRNA transcripts for further validation by droplet digital PCR (ddPCR) assays, we used miRNA-seq data from a small technical feasibility pilot study that we had previously conducted on exosome isolates from saliva (2 mL) collected from 5 patients with incident primary HNSCC (obtained prior to initiation of treatment) and 5 cancer-free controls (see Table 3 for a description of clinical-demographic characteristics). The number of sequencing reads for each sample ranged from 1.6 million to 27.4 million (median = 12.4 million). There were a total of 1,334 mature miRNA transcripts detected across samples, with 307 transcripts (12%) detected solely in salivary exosomes from cases. We further evaluated the 8 candidate miRNA that were secreted solely and universally by the HNSCC cell lines (i.e. not secreted by the oral epithelial control cells). In particular, *miR-486-5p* and *miR-486-3p* showed considerable promise, with 2/5 of cases expressing drastically higher levels of these transcripts relative to controls (Figure 5A). We also performed a *post-hoc* assessment of *miRNA-10b-5p* since, despite the small sample size of the pilot study, we observed significant differential secretion (p = 0.006), with transcripts present at relatively high levels in salivary exosomes from 3 of the 5 cases but none detected in those from the 5 controls (Figure 5B). Upon further review of our *in vitro* data, we noted that it was significantly upregulated in exosomes derived from 3 of 4 HNSCC (Q < 0.1) and nominally significant in the 4^th^ (p_undajusted_ = 0.04). When either *miR-486-5p* or *miR-486-3p* was combined with *miR-10b-5p*, the substantial separation of these markers could clearly distinguish 80% (4/5) of the HNSCC cases from controls.

**Table 3:** Description of head and neck squamous cell carcinoma cases and healthy controls from the miRNA-sequencing pilot and droplet digital PCR (ddPCR) saliva studies

	miRNA-seq pilot			ddPCR assays		
	Cases (n = 5)	Controls (n = 5)	p_difference_	Cases (n = 11)	Controls (n = 9)	p_difference_
Age, median years (range)	63 (50-76)	38 (29-66)	0.05^a^	58 (47-73)	36 (19-53)	0.0003^a^
Sex, n (%)						
Female	1 (20%)	3 (60%)	0.52^b^	2 (18%)	5 (56%)	0.16^b^
Male	4 (80%)	2 (40%)		9 (82%)	4 (44%)	
Race, n (%)						
Caucasian	5 (100%)	5 (100%)	> 0.99^b^	11 (100%)	5 (56%)	0.03^b^
Black/African American	0 (0%)	0 (0%)		0 (0%)	3 (33%)	
Other	0 (0%)	0 (0%)		0 (0%)	1 (11%)	
Smoking status, n (%)						
Never	0 (0%)	5 (100%)	0.02^b^	2 (18%)	6 (75%)	0.04^b^
Former	2 (40%)	0 (0%)		7 (64%)	1 (13%)	
Current	1 (20%)	0 (0%)		2 (18%)	1 (13%)	
Missing	2 (40%)	0 (0%)				
Alcohol status						
Non-drinker	1 (20%)	0 (0%)	0.38^b^	5 (45%)	3 (38%)	> 0.99^b^
Drinker	2 (40%)	5 (100%)		6 (55%)	5 (63%)	
Missing	2 (40%)	0 (0%)		0 (0%)	1 (11%)	
Primary tumor site, n (%)						
Oral cavity	3 (60%)	---		3 (27%)	---	
Oropharynx	2 (40%)	---		8 (73%)	---	
Stage at diagnosis, n (%)						
Early (stage I or II)	2 (40%)	---		1 (9%)	---	
Advanced (stage III or IV)	3 (60%)	---		10 (91%)	---	
p16 immunohistochemistry, n (%)						
Negative	1 (20%)	---		1 (9%)	---	
Positive	1 (20%)	---		8 (73%)	---	
Not evaluated^c^	3 (60%)	---		2 (18%)	---	

^a^ Wilcoxon rank-sum test.

^b^ Fishers's exact test.

^c^ All tumors that were not evaluated for p16 originated in the oral cavity.

#### Droplet digital PCR for *miR-486-5p* and *miR-10b-5p*

On the basis of the miRNA-seq pilot data, we selected *miR-486-5p* and *miR-10b-5p* for further validation by ddPCR in an independent set of saliva samples from an additional 11 cases and 9 cancer-free controls (see Table 3 for a description of clinical-demographic characteristics). *miR-10b-5p* was detected at very low levels (if at all) in the majority of samples but had substantially higher levels in 3/11 cases (all originating in the oral cavity) and 0/9 controls (Figure 5C). The respective median concentration and inter-quartile ranges for cases and controls were 0.49 copies/μL (IQR: 0.1-1.8 copies/μL) vs. 0.20 copies/μL (IQR: 0-0.38 copies/μL). Setting a cutpoint > 1.0 copies/μL would correspond to a sensitivity = 18% and specificity = 100%.

*miR-486-5p* was detected in salivary exosomes from all samples but was substantially elevated in 5/11 cases, including 2 p16-positive oropharyngeal cases, and 1/9 controls (Figure 5D). The respective median concentration and inter-quartile ranges for cases and controls were 34.6 copies/μL (IQR: 9.5-462 copies/μL) and 9.4 copies/μL (7.1-29.8 copies/μL). Setting a conservative cutpoint > 100 copies/μL (the highest concentration among controls was 37.3 copies/μL) would correspond to a sensitivity = 45% and specificity = 89%. Importantly, the *miR-486-5p* assay was able to identify the lone early stage case (stage I), which is encouraging from the viewpoint of early detection.

## DISCUSSION

We have demonstrated clear patterns of exosomal miRNA secretion that distinguish HNSCC cells from primary oral epithelial cells derived from healthy donors. Several miRNA were differentially secreted solely by HNSCC cells, thereby yielding a candidate set of exosomal miRNA for further assessment as HNSCC biomarkers, and providing new insight into tumor-derived exosome biology. Importantly, several of the candidate exosomal miRNA (*miR-486-5p*, *miR-486-3p*, and *miR-10b-5p*) were detectable at substantially higher levels in saliva for a subset of HNSCC patients relative to cancer-free controls, highlighting the potential clinical utility of exosomal miRNA as non-invasive salivary biomarkers. This latter finding is important since there are very few human studies in the literature that evaluated biomolecular cargo of salivary exosomes as cancer biomarkers [24–26]. While one of the aforementioned studies examined protein cargo of salivary exosomes as biomarkers of oral squamous cell carcinoma [26], we are aware of no studies evaluating miRNA (or any other form of RNA) as biomarkers of HNSCC.

Several themes emerged with respect to pathway enrichment among the common subset of differentially secreted exosomal miRNA. One observation was the enrichment for miRNA negatively regulating genes involved in immune pathways, including a number of targets involving toll-like receptors (TLR) or Fc receptor pathways, which play important roles in cancer surveillance through immune stimulation [27, 28]. These findings suggest a novel exosome-driven mechanism through which the tumor can manipulate its microenvironment to evade immune surveillance and destruction [29], and may offer insights into innovative avenues for immunotherapy and potential markers for predicting its efficacy. This is also consistent with reports that alterations of TLR pathways in the tumor microenvironment can contribute to therapeutic resistance and progression in solid tumors [30, 31], including HNSCC [32, 33]. Further, it has been demonstrated that exosomal-secretion of miRNA can affect the tumor microenvironment through interactions with TLR8 in non-small cell lung cancer [34] and neuroblastoma [35], which was implicated in cisplatin-resistance for the latter [35]. Secondly, we observed significant enrichment for exosomal miRNA targeting a sizable number of biological processes relating to metabolism/catabolism/synthesis, including glucose transport, glutamate secretion, nitrogen compound metabolism, insulin secretion, and energy metabolism. Reprograming of cellular metabolism, which was first described by Otto Warburg in 1956 [36], is considered a hallmark of cancer [29]. Malignant cells, including HNSCC [37], have a voracious appetite for glucose and glutamine, which are required to support their high energy and biosynthesis demands due to a predilection for aerobic glycolysis [38, 39]. Of particular significance is that *miR-122*, which was detected solely and universally in exosomes from HNSCC cell lines, has been reported to suppress glucose uptake in surrounding cells when secreted from breast cancer cells via microvesicles, thereby selectively increasing glucose availability for cancer cells and promoting metastasis [40].

Among the strengths of this work were the inclusion of primary oral epithelial cells pooled from healthy donors for controls, the use of a diverse set of 4 HNSCC cell lines with each cultured in triplicate, employment of miRNA-sequencing which allowed for comprehensive interrogation of exosomal miRNA cargo and cellular expression profiles without the need to rely on the availability or sensitivity of microarray probes, and replication/translation in saliva from HNSCC patients and healthy controls. Moreover, the use of NTA, TEM, and Western blot for exosome characterization provides confidence that we have selectively isolated small extracellular vesicles within the exosome size range. Furthermore, the use of differential ultracentrifugation is not only the current gold standard but also avoids any potential biases that may accompany commercial affinity-based approaches in isolating cancer-associated exosomes [41]. One potential limitation is the comparison of exosomes from established stable cancer cell lines to those from primary epithelial cells. While it is conceivable that some of the differences in exosomal cargo could stem from this distinction, we highlight the observation that many, if not most, of the differentially secreted miRNA were cancer-associated or involved in manipulation of cellular processes that are key players in cancer cell survival or progression. We also acknowledge that the 4 cell lines used herein do not fully represent the entire complex and heterogeneous spectrum of HNSCC, particularly given that all 4 cell lines were HPV-negative. However, the cell lines used in this study were selected to represent HNSCC from a diverse set of sites (buccal, tongue, oropharynx, hypopharynx), and, despite the modest variety of cell lines used, clear cancer-specific patterns of exosomal miRNA secretion have emerged. Importantly, we have detected some of these markers at higher levels in saliva from patients with HNSCC relative to healthy controls, highlighting the downstream clinical potential.

This work yields a novel set of candidate exosomal miRNA that may have utility as non-invasive salivary biomarkers of HNSCC. While the sensitivity was relatively low for each individual miRNA tested, the specificity was high, highlighting the strong potential as part of multi-marker panels and underscoring the need for further testing in larger human cohorts. It also provides important insight into tumor biology and intercellular cross-talk within the tumor microenvironment: in addition to furnishing further evidence for a role in manipulating the inflammatory milieu, it has underscored a potential role for exosomal transfer of miRNA for the purpose of manipulating the metabolic state of surrounding cells to promote tumor cell survival, tumor growth, and metastatic potential. Therefore it remains essential to continue elucidation of the significance and impact of exosomal signaling within the tumor microenvironment as well as systemically at metastatic sites through further epidemiologic and experimental studies. Development of novel biomarkers to aid in the early detection and diagnosis of head and neck cancer is of paramount importance towards reducing the devastating morbidity and mortality toll of this disease.

## MATERIALS AND METHODS

We cultured 4 discrete commercially-available HNSCC cell lines that originated from 4 different sites in the upper aerodigestive tract: [1] H413 (buccal; Sigma-Aldrich, *St. Louis, MO*; authenticated November 24, 2006) [14]; [2] Detroit 562 (pharynx metastatic to pleura; obtained May 7, 2016 from ATCC, *Manassas, VA*; authenticated February 17, 2015) [15]; [3] FaDu (hypopharynx; obtained May 7, 2016 from ATCC; authenticated February 27, 2014) [16]; and [4] Cal 27 (tongue; obtained May 7, 2016 from ATCC; authenticated November 26, 2014) [17]. ATCC cell lines were authenticated by ATCC through morphological assessment, cytochrome C oxidase subunit I (COI) DNA barcoding, and short tandem repeat (STR) analysis; cells were tested for Mycoplasma contamination using the Hoechst staining and agar culture methods. Sigma-Aldrich obtained the H413 cell line from the European Collection of Authenticated Cell Cultures (ECACC); cells were authenticated using STR analysis and tested for Mycoplasma contamination using the Hoechst staining, agar culture, and Mycoplasma-specific PCR. We additionally obtained primary human gingival epithelial cells that were pooled from 3 healthy female donors (HGEPp; CELLnTEC, *Bern, Switzerland*) for comparison; cells were tested for contamination using Mycoplasma-specific RT-PCR.

### Cell culture conditions

Cells were cultivated in supplier-recommended media with 10% fetal bovine serum (FBS) that was super-depleted of exosomes via 18 hour ultracentrifugation at 100,000×*g* (verified via nanoparticle tracking analysis) and 1% penicillin/streptomycin at 37°C with 5% CO_2_ in 150 cm^2^ flasks with 25mL media. Briefly, H413 was grown in Dulbecco's Modified Eagle's Medium/Nutrient F-12 Ham (DMEM-F12; Sigma-Aldrich), Detroit 562 and FaDu were grown in Eagle’s Minimum Essential Medium (MEM; ATCC), and Cal 27 was grown in Dulbecco’s Modified Eagle’s Medium (DMEM; ATCC); the pooled set of primary non-pathologic oral epithelial cells were cultured in fully-defined CnT-Prime media (CELLnTEC), which is completely free of animal or human- derived components, and 1% penicillin/streptomycin/Fungizone, at 37°C with 5% CO_2_. To achieve adequate volume for exosome isolation, cells were cultured in 2-pair sets of flasks in triplicate (6 flasks total per cell-line). Culture media were replaced approximately 48 hours prior to cells reaching 80-90% confluence; media and cells were respectively collected after 48 hours. The media from each 2-flask pair were combined (50 mL total). After media was harvested, cells were detached from each flask and respectively pooled for each cell line.

### Exosome isolation from conditioned media

Exosomes were isolated and purified from the conditioned cell culture media by differential ultracentrifugation, according to the protocol described by Gallo et al. [18]. Briefly: each cell culture media sample was spun down at 300×*g* for 10 minutes (4°C), followed by 2,000×*g* for 20 min (4°C) to eliminate dead cells, then 10,000×*g* for another 30 min (4°C) to remove debris. The media was then pelleted by ultracentrifugation at 100,000×*g* for 70 min at 4°C; the supernatant was discarded and the pellet was re-suspended in 1 mL phosphate-buffered saline (PBS) and centrifuged again at 100,000×*g* for 70 min. The supernatant was discarded and the pellet was re-suspended in 200μL PBS and stored at -80°C until further analysis.

### Exosome characterization

The presence of purified exosomes was verified via nanoparticle tracking analysis (NTA) using a NanoSight NS300 instrument (Malvern, *Worcestershire, UK*). Additionally, visual confirmation was performed with a JEOL JEM-1230 transmission electron microscope (TEM), using the methods described by Théry et al [42]; and Western blot analysis was performed for exosome associated tetraspanin CD81 and cytosolic endosomal sorting complex component TSG101 [19]. Westerns were run using 12% polyacrylamide gels in a mini gel tank (Thermo Fisher Scientific, *Waltham, MA*) with 10× Tris/Glycine/SDS Buffer. Samples were mixed with 4x Laemmli SDS sample buffer (non-reducing) and proteins were subsequently transferred onto PVDF-membrane with Step 1- Transfer buffer on a Pierce Power Station (Thermo Fisher Scientific) for at 1.3A constant. CD81 (ab79559, Abcam, *Cambridge, UK*) and TSG101 (ab30871, Abcam) antibody was added 1:1000 in 5% Milk and 5% BSA, respectively, in TBST overnight at 4°C. After washing the membrane 3 × 5 minutes in TBST, secondary antibody Goat anti-Mouse IgG H&L (ab205719, Abcam) was added 1:3000 in 5% Milk in TBST for three hours for CD81 and secondary antibody Goat anti-Rabbit IgG H&L (ab205718, Abcam) was added 1:2000 in 5% BSA in TBST for two hours, respectively. Following 3 final washes at 5 minutes each, detection was performed using a WesternBright ECL detection kit (Advansta, *Menlo Park, CA*) on a C-DiGit Blot Scanner (LI-COR Biotechnology, *Lincoln, NE*).

### RNA extraction

Total RNA was extracted from each exosome pellet using the miRNeasy Micro kit (Qiagen, *Valencia, CA*) according to the manufacturer’s suggested protocol; total RNA was also extracted from the pooled cells for each respective cell line using the same kit, with 7.5 x10^5^ cells in each reaction. Total RNA concentrations were initially evaluated using a NanoDrop 2000 (Thermo Fisher Scientific) and confirmed by Qubit fluorometer (Thermo Fisher Scientific).

### MicroRNA-sequencing

MicroRNA-sequencing (miRNA-seq) was performed by the University of Cincinnati Genomics, Epigenomics and Sequencing Core (*Cincinnati, OH*). Library preparation was performed using the NEBNext Multiplex Small RNA Library Prep kit (NEB, *Ipswich, MA*) with 20 ng to 1 μg of total RNA in a 6-μL solution as input, following the manufacturer’s protocol with modification of library size selection. This protocol takes advantage of the natural structure common to most known miRNA molecules. In brief, the RNA 3' adaptor was specifically ligated to miRNA with the excess adaptor removed by hybridization. The 5’ ends of miRNA were then ligated to the 5’ adaptor, followed by reverse transcription to convert the ligated small RNA into cDNA, which was then uniquely indexed by PCR to generate the sequencing library. The miRNA concentration of the library was evaluated by Bioanalyzer (Agilent Technologies, *Santa Clara, CA*). Based on this measurement, the libraries were equal-molar pooled (up to 24 samples with different barcodes). The pooled samples were then mixed with DNA ladder containing specific sizes (135 and 155 bp) for precise positioning and recovery in a 3% agarose gel electrophoresis, and libraries ranging from 135 to 155 bp were gel purified and eluted in 18 μL. The ladder in the mixture improves library recovery and does not interfere sequencing (data not shown). After gel purification, 2 μL of the libraries were 1:10^4^ diluted in dilution buffer (10 mM Tris-HCl, pH 8.0 with 0.05% Tween 20) and analyzed with the Kapa Library Quantification kit (Kapa Biosystems, *Wilmington, MA*) using an ABI 9700HT Fast Real-Time PCR system (Thermo Fisher Scientific). The quantified libraries were clustered onto a flow cell at the concentration of 10 pM using the TruSeq SR Cluster Kit v3 (Illumina), and sequenced for 50 cycles using TruSeq SBS kit on a HiSeq system (Illumina).

### Pre-processing and sequence alignment

Sequence reads were pre-processed to remove adapters and retain only 16-30 base pair- length reads. Reads were aligned to the reference human genome (hg19) using the Bowtie aligner [43]. The reads aligning to each known mature miRNA were counted using Bioconductor packages for next-generation sequencing data analysis [44] based on miRNA definitions in miRBase database [45].

### Statistical analysis

The differential expression analysis between different sample types was performed using the negative binomial statistical model of read counts as implemented in the *DESeq* Bioconductor package [46]. The statistical significance of differential expression is established based on the FDR-adjusted p-values [47]. The cluster analysis of all differentially expressed miRNA was performed using the Bayesian infinite mixture model [48]. Pathway enrichment analysis was conducted with DIANA mirPath v.3 [21] for miRNA that were differentially expressed in exosomes derived from all 4 head and neck cancer cell lines. Analysis was based on both experimentally supported and putative gene targets predicted *in silico*, annotated in DIANA-TarBase v.7.0 [22] and DIANA-microT-CDS [23], respectively, and was performed for both Kyoto Encyclopedia of Genes and Genomes (KEGG) pathways [49] and Gene Ontology (GO) [50]. Enrichment p-values were corrected for false discovery rate (FDR) [51], and were considered significant when adjusted p ≤ 0.05.

### Assessment of candidate exosomal miRNA in human saliva samples

#### MicroRNA-sequencing (miRNA-seq) pilot

To preliminarily assess the potential clinical utility of the 8 candidate miRNA that were solely and universally differentially secreted by the 4 HNSCC cell lines in a non-invasive biofluid and guide the selection of miRNA transcripts for further validation by droplet digital PCR (ddPCR) assays, we used miRNA-seq data from a small technical feasibility pilot study that we had previously conducted. Saliva samples were obtained from 5 patients with a newly diagnosed initial primary HNSCC (pre-treatment) and 5 cancer-free control subjects. Exosomes were isolated and purified according to the protocol described by Gallo et al. [18]. Briefly, each saliva sample was centrifuged at 1,500×*g* for 10 minutes to pull down any cells or cellular debris, after which the resultant supernatant was centrifuged at 17,000×*g* for 15 minutes to pull down any smaller debris or cellular organelles. The supernatant was then spun in a L8-60M ultracentrifuge (Beckman Coulter, *Brea, CA*) using a 45Ti fixed rotor at 160,000 ×*g* for 1 hour, resulting in a pellet of salivary exosomes. Total RNA was extracted from the exosome pellets using the miRNeasy Micro kit (Qiagen, *Valencia, CA*) according to the manufacturer’s suggested protocol and miRNA-seq was performed as described above.

#### Droplet digital PCR (ddPCR)

On the basis of the miRNA-seq pilot data, we selected 2 miRNA transcripts for further validation via ddPCR in an independent set of saliva samples from an additional 11 cases and 9 controls. TaqMan miRNA assays (Thermo Fisher Scientific) for were obtained for the respective mature miRNA sequences. Exosomal miRNA was converted to cDNA using the TaqMan MicroRNA Reverse Transcription Kit (Thermo Fisher Scientific) according to the manufacturer’s suggested protocol. Each sample (20 °L aliquot) will be combined with ddPCR supermix for probes (Bio-Rad, *Hercules, CA*) and then partitioned into 15,000-20,000 droplets, resulting in a random distribution of sample in each, using a QX100 droplet generator (Bio-Rad). Emulsified samples were transferred to a 96-well plate and heat-sealed with foil, and PCR was performed (95°C for 10 min -> 40 cycles of 95°C for 15 s and 60°C for 1 min -> 98°C for 10 min) using a C1000 Touch deep-well thermocycler (Bio Rad). Amplified droplets were loaded on a QX100 droplet reader (Bio Rad) and analyzed using QuantaSoft software (Bio Rad). No template controls were included on each plate for each assay to monitor the level of background signal; a no amplication control was also included for each assay to monitor the presence of contaminating DNA in the sample.

This study was approved by the University of Cincinnati Institutional Review Board; all subjects provided written informed consent for participation in this study.

## SUPPLEMENTARY MATERIALS TABLES




