# Supplementary material for: Comprehensive microRNA-sequencing of exosomes derived from head and neck carcinoma cells in vitro reveals common secretion profiles and potential utility as salivary biomarkers
Source: Oncotarget. 2017 Jul 27;8(47):82459–74. doi: 10.18632/oncotarget.19614 (PMC5669904; doi:10.18632/oncotarget.19614)
Supplement: Supplementary file 1 [file oncotarget-08-82459-s001.pdf]

Comprehensive microRNA-sequencing of exosomes derived from head and neck carcinoma cells *in vitro* reveals common secretion profiles and potential utility as salivary biomarkers

SUPPLEMENTARY MATERIALS

Supplemenatry Table 1: Differentially secreted miRNA for HNSCC cell lines relative to primary non-pathologic oral epithelial control cells (Q < 0.1)

See Supplemenatry File 1

Supplementary Table 2: KEGG pathways that were enriched among experimentally supported (TarBase) targets of miRNA that were differentially secreted by all 4 HNSCC cell lines relative to primary non-pathologic oral epithelial control cells

See Supplemenatry File 1

Supplementary Table 3: KEGG pathways that were enriched among predicted (MicroT-CDS) targets of miRNA that were differentially secreted by all 4 HNSCC cell lines relative to primary non-pathologic oral epithelial control cells

See Supplemenatry File 1

Supplementary Table 4: GO categories that were enriched among experimentally supported (TarBase) targets of miRNA that were differentially secreted by all 4 HNSCC cell lines relative to primary non-pathologic oral epithelial control cells

See Supplemenatry File 1

Supplementary Table 5: GO categories that were enriched among predicted (MicroT-CDS) targets of miRNA that were differentially secreted by all 4 HNSCC cell lines relative to primary non-pathologic oral epithelial control cells

See Supplemenatry File 1
