# Supplementary material for: Comprehensive microRNA-sequencing of exosomes derived from head and neck carcinoma cells in vitro reveals common secretion profiles and potential utility as salivary biomarkers
Source: Oncotarget. 2017 Jul 27;8(47):82459–74. doi: 10.18632/oncotarget.19614 (PMC5669904; doi:10.18632/oncotarget.19614)
Supplement: Supplementary file 2 [file oncotarget-08-82459-s002.docx]

**TABLE OF CONTENTS**

|  | Page(s) | Description |
| --- | --- | --- |
| Table S1 | 2-7 | Differentially secreted miRNA for HNSCC cell lines relative to primary non-pathologic oral epithelial control cells (Q < 0.1). |
| Table S2 | 8-9 | KEGG pathways that were enriched among experimentally supported (TarBase) targets of miRNA that were differentially secreted by all 4 HNSCC cell lines relative to primary non-pathologic oral epithelial control cells. |
| Table S3 | 10-11 | KEGG pathways that were enriched among predicted (MicroT-CDS) targets of miRNA that were differentially secreted by all 4 HNSCC cell lines relative to primary non-pathologic oral epithelial control cells. |
| Table S4 | 12-15 | GO categories that were enriched among experimentally supported (TarBase) targets of miRNA that were differentially secreted by all 4 HNSCC cell lines relative to primary non-pathologic oral epithelial control cells. |
| Table S5 | 16-18 | GO categories that were enriched among predicted (MicroT-CDS) targets of miRNA that were differentially secreted by all 4 HNSCC cell lines relative to primary non-pathologic oral epithelial control cells. |

**Supplementary Table 1: Differentially secreted miRNA for HNSCC cell lines relative to primary non-pathologic oral epithelial control cells (Q < 0.1)**

| **Cell Line** | **geneid** | **symbol** | **log2FoldChange** | **FDR-adj P-value** |
| --- | --- | --- | --- | --- |
| FaDu | MIMAT0001631 | hsa-miR-451;hsa-miR-451a; | Inf | 7.52E-52 |
| Cal 27 | MIMAT0004749 | hsa-miR-424*;hsa-miR-424-3p; | -6.755281275 | 1.78E-48 |
| Cal 27 | MIMAT0000421 | hsa-miR-122a;hsa-miR-122;hsa-miR-122-5p; | Inf | 2.04E-48 |
| Cal 27 | MIMAT0019877 | hsa-miR-3591-3p; | Inf | 2.04E-48 |
| FaDu | MIMAT0004749 | hsa-miR-424*;hsa-miR-424-3p; | -6.514593768 | 1.66E-41 |
| Detroit 562 | MIMAT0004749 | hsa-miR-424*;hsa-miR-424-3p; | -5.678040896 | 3.87E-41 |
| Cal 27 | MIMAT0002177 | hsa-miR-486;hsa-miR-486-5p; | Inf | 5.82E-35 |
| Cal 27 | MIMAT0004762_1 |  | Inf | 5.82E-35 |
| Cal 27 | MIMAT0001631 | hsa-miR-451;hsa-miR-451a; | Inf | 4.47E-30 |
| Cal 27 | MIMAT0000445 | hsa-miR-126;hsa-miR-126-3p; | 4.391251751 | 9.63E-30 |
| FaDu | MIMAT0000093 | hsa-miR-93;hsa-miR-93-5p; | 4.951573173 | 1.30E-27 |
| Cal 27 | MIMAT0002177_1 |  | Inf | 6.03E-27 |
| Cal 27 | MIMAT0004762 | hsa-miR-486-3p; | Inf | 6.03E-27 |
| FaDu | MIMAT0000421 | hsa-miR-122a;hsa-miR-122;hsa-miR-122-5p; | Inf | 1.47E-26 |
| FaDu | MIMAT0019877 | hsa-miR-3591-3p; | Inf | 1.47E-26 |
| Detroit 562 | MIMAT0000646 | hsa-miR-155;hsa-miR-155-5p; | 5.421828245 | 2.02E-26 |
| FaDu | MIMAT0002177 | hsa-miR-486;hsa-miR-486-5p; | Inf | 3.56E-26 |
| FaDu | MIMAT0004762_1 |  | Inf | 3.56E-26 |
| Detroit 562 | MIMAT0000077 | hsa-miR-22;hsa-miR-22-3p; | 1.985697465 | 2.36E-23 |
| Detroit 562 | MIMAT0000421 | hsa-miR-122a;hsa-miR-122;hsa-miR-122-5p; | Inf | 2.61E-22 |
| Detroit 562 | MIMAT0019877 | hsa-miR-3591-3p; | Inf | 2.61E-22 |
| FaDu | MIMAT0000098 | hsa-miR-100;hsa-miR-100-5p; | -3.799004171 | 9.18E-22 |
| Detroit 562 | MIMAT0000093 | hsa-miR-93;hsa-miR-93-5p; | 4.578260193 | 1.04E-21 |
| Detroit 562 | MIMAT0000081 | hsa-miR-25;hsa-miR-25-3p; | 4.284440314 | 1.16E-21 |
| FaDu | MIMAT0000435 | hsa-miR-143;hsa-miR-143-3p; | Inf | 1.36E-21 |
| FaDu | MIMAT0002177_1 |  | Inf | 2.35E-20 |
| FaDu | MIMAT0004762 | hsa-miR-486-3p; | Inf | 2.35E-20 |
| Cal 27 | MIMAT0000435 | hsa-miR-143;hsa-miR-143-3p; | Inf | 4.51E-19 |
| Detroit 562 | MIMAT0000063 | hsa-let-7b;hsa-let-7b-5p; | -2.265550872 | 5.61E-19 |
| H413 | MIMAT0000253 | hsa-miR-10a;hsa-miR-10a-5p; | 4.439572197 | 2.71E-18 |
| Cal 27 | MIMAT0000098 | hsa-miR-100;hsa-miR-100-5p; | -3.078775095 | 2.83E-18 |
| FaDu | MIMAT0000081 | hsa-miR-25;hsa-miR-25-3p; | 4.212751746 | 6.85E-18 |
| Detroit 562 | MIMAT0001631 | hsa-miR-451;hsa-miR-451a; | Inf | 1.47E-17 |
| Cal 27 | MIMAT0000081 | hsa-miR-25;hsa-miR-25-3p; | 4.057299895 | 1.82E-16 |
| H413 | MIMAT0000421 | hsa-miR-122a;hsa-miR-122;hsa-miR-122-5p; | Inf | 2.28E-15 |
| H413 | MIMAT0019877 | hsa-miR-3591-3p; | Inf | 2.28E-15 |
| Detroit 562 | MIMAT0000087 | hsa-miR-30a-5p;hsa-miR-30a;hsa-miR-30a-5p; | 2.642393879 | 9.71E-15 |
| FaDu | MIMAT0000280 | hsa-miR-223;hsa-miR-223-3p; | Inf | 2.17E-14 |
| H413 | MIMAT0000078 | hsa-miR-23a;hsa-miR-23a-3p; | 3.007177154 | 3.04E-14 |
| FaDu | MIMAT0000451 | hsa-miR-150;hsa-miR-150-5p; | Inf | 1.02E-13 |
| Detroit 562 | MIMAT0000062_2 |  | -2.239443471 | 1.28E-13 |
| Cal 27 | MIMAT0000646 | hsa-miR-155;hsa-miR-155-5p; | 4.584328742 | 1.35E-13 |
| Cal 27 | MIMAT0000063 | hsa-let-7b;hsa-let-7b-5p; | -2.264682882 | 1.38E-13 |
| Detroit 562 | MIMAT0000062_1 |  | -2.208769731 | 2.04E-13 |
| Cal 27 | MIMAT0000451 | hsa-miR-150;hsa-miR-150-5p; | Inf | 3.98E-13 |
| Cal 27 | MIMAT0000087 | hsa-miR-30a-5p;hsa-miR-30a;hsa-miR-30a-5p; | 2.572853967 | 6.43E-13 |
| Detroit 562 | MIMAT0000682 | hsa-miR-200a;hsa-miR-200a-3p; | 3.29237444 | 4.83E-12 |
| Cal 27 | MIMAT0000280 | hsa-miR-223;hsa-miR-223-3p; | Inf | 1.00E-11 |
| FaDu | MIMAT0000445 | hsa-miR-126;hsa-miR-126-3p; | 4.531103472 | 2.58E-11 |
| Cal 27 | MIMAT0000093 | hsa-miR-93;hsa-miR-93-5p; | 3.643822814 | 5.16E-11 |
| H413 | MIMAT0000063 | hsa-let-7b;hsa-let-7b-5p; | -3.006138407 | 7.82E-11 |
| H413 | MIMAT0000093 | hsa-miR-93;hsa-miR-93-5p; | 3.768095517 | 1.58E-10 |
| Detroit 562 | MIMAT0000318 | hsa-miR-200b;hsa-miR-200b-3p; | 2.077726069 | 2.02E-10 |
| Detroit 562 | MIMAT0000449 | hsa-miR-146;hsa-miR-146a;hsa-miR-146a-5p; | 4.331778215 | 2.71E-10 |
| H413 | MIMAT0000081 | hsa-miR-25;hsa-miR-25-3p; | 3.616468846 | 8.49E-10 |
| Detroit 562 | MIMAT0000256_1 |  | 1.871439491 | 9.03E-10 |
| H413 | MIMAT0000617 | hsa-miR-200c;hsa-miR-200c-3p; | 1.924816683 | 1.82E-09 |
| Detroit 562 | MIMAT0000098 | hsa-miR-100;hsa-miR-100-5p; | -2.013116843 | 2.05E-09 |
| Cal 27 | MIMAT0000689 | hsa-miR-99b;hsa-miR-99b-5p; | -2.620643941 | 3.90E-09 |
| H413 | MIMAT0000318 | hsa-miR-200b;hsa-miR-200b-3p; | 2.322336935 | 4.38E-09 |
| FaDu | MIMAT0004614 | hsa-miR-193a-5p; | -6.519939907 | 4.76E-09 |
| H413 | MIMAT0004749 | hsa-miR-424*;hsa-miR-424-3p; | -5.09171905 | 6.98E-09 |
| Detroit 562 | MIMAT0000078 | hsa-miR-23a;hsa-miR-23a-3p; | 2.167063588 | 1.31E-08 |
| Detroit 562 | MIMAT0000064 | hsa-let-7c;hsa-let-7c-5p; | -2.585709556 | 1.43E-08 |
| H413 | MIMAT0002177 | hsa-miR-486;hsa-miR-486-5p; | Inf | 1.50E-08 |
| H413 | MIMAT0004762_1 |  | Inf | 1.50E-08 |
| Detroit 562 | MIMAT0004614 | hsa-miR-193a-5p; | -8.329067522 | 1.67E-08 |
| Detroit 562 | MIMAT0000435 | hsa-miR-143;hsa-miR-143-3p; | Inf | 2.33E-08 |
| FaDu | MIMAT0000420 | hsa-miR-30b;hsa-miR-30b-5p; | 5.813532024 | 2.97E-08 |
| Detroit 562 | MIMAT0005951 | hsa-miR-1307;hsa-miR-1307-3p; | -2.035184235 | 5.45E-08 |
| FaDu | MIMAT0000245 | hsa-miR-30d;hsa-miR-30d-5p; | 1.800096651 | 6.33E-08 |
| H413 | MIMAT0002177_1 |  | Inf | 7.19E-08 |
| H413 | MIMAT0004762 | hsa-miR-486-3p; | Inf | 7.19E-08 |
| FaDu | MIMAT0000083 | hsa-miR-26b;hsa-miR-26b-5p; | 4.021282541 | 1.14E-07 |
| FaDu | MIMAT0002874 | hsa-miR-503;hsa-miR-503-5p; | -4.677692292 | 1.14E-07 |
| FaDu | MIMAT0019022 | hsa-miR-4488; | -6.691232167 | 1.40E-07 |
| Cal 27 | MIMAT0019022 | hsa-miR-4488; | -6.381111892 | 1.55E-07 |
| FaDu | MIMAT0000617 | hsa-miR-200c;hsa-miR-200c-3p; | 1.618331877 | 1.72E-07 |
| Detroit 562 | MIMAT0000757 | hsa-miR-151;hsa-miR-151-3p;hsa-miR-151a-3p; | 2.183952665 | 1.74E-07 |
| FaDu | MIMAT0000254 | hsa-miR-10b;hsa-miR-10b-5p; | 6.291379607 | 2.13E-07 |
| Detroit 562 | MIMAT0000445 | hsa-miR-126;hsa-miR-126-3p; | 2.775913321 | 2.37E-07 |
| Detroit 562 | MIMAT0030019 | hsa-miR-7704; | -5.227272147 | 2.86E-07 |
| Cal 27 | MIMAT0000254 | hsa-miR-10b;hsa-miR-10b-5p; | 6.239095503 | 3.27E-07 |
| H413 | MIMAT0000445 | hsa-miR-126;hsa-miR-126-3p; | 2.891581238 | 3.91E-07 |
| Cal 27 | MIMAT0002874 | hsa-miR-503;hsa-miR-503-5p; | -4.307456841 | 4.52E-07 |
| Cal 27 | MIMAT0000062_2 |  | -1.711932206 | 6.86E-07 |
| Cal 27 | MIMAT0000318 | hsa-miR-200b;hsa-miR-200b-3p; | 1.8960137 | 6.86E-07 |
| FaDu | MIMAT0000423_1 |  | -2.710850256 | 8.18E-07 |
| FaDu | MIMAT0000279 | hsa-miR-222;hsa-miR-222-3p; | -2.18414783 | 8.96E-07 |
| Detroit 562 | MIMAT0002177 | hsa-miR-486;hsa-miR-486-5p; | Inf | 9.53E-07 |
| Detroit 562 | MIMAT0004762_1 |  | Inf | 9.53E-07 |
| Cal 27 | MIMAT0000682 | hsa-miR-200a;hsa-miR-200a-3p; | 2.526367835 | 1.05E-06 |
| Detroit 562 | MIMAT0000244 | hsa-miR-30c;hsa-miR-30c-5p; | 2.749119048 | 1.19E-06 |
| Detroit 562 | MIMAT0000617 | hsa-miR-200c;hsa-miR-200c-3p; | 1.202895147 | 1.55E-06 |
| FaDu | MIMAT0000244 | hsa-miR-30c;hsa-miR-30c-5p; | 2.792978829 | 2.14E-06 |
| Cal 27 | MIMAT0000423_1 |  | -2.632528802 | 2.41E-06 |
| Cal 27 | MIMAT0000062_1 |  | -1.614036037 | 2.51E-06 |
| Cal 27 | MIMAT0000436 | hsa-miR-144;hsa-miR-144-3p; | Inf | 4.57E-06 |
| H413 | MIMAT0000682 | hsa-miR-200a;hsa-miR-200a-3p; | 2.593121308 | 4.63E-06 |
| H413 | MIMAT0000418 | hsa-miR-23b;hsa-miR-23b-3p; | 3.189544038 | 4.64E-06 |
| H413 | MIMAT0001631 | hsa-miR-451;hsa-miR-451a; | Inf | 4.64E-06 |
| H413 | MIMAT0004977 | hsa-miR-934; | Inf | 4.68E-06 |
| FaDu | MIMAT0000440 | hsa-miR-191;hsa-miR-191-5p; | 1.654122493 | 5.44E-06 |
| Detroit 562 | MIMAT0019022 | hsa-miR-4488; | -5.837154691 | 5.47E-06 |
| FaDu | MIMAT0000456 | hsa-miR-186;hsa-miR-186-5p; | 3.517787342 | 5.70E-06 |
| Detroit 562 | MIMAT0000455 | hsa-miR-185;hsa-miR-185-5p; | 3.905693922 | 5.72E-06 |
| Cal 27 | MIMAT0000244 | hsa-miR-30c;hsa-miR-30c-5p; | 2.6993664 | 5.93E-06 |
| Detroit 562 | MIMAT0002177_1 |  | Inf | 5.94E-06 |
| Detroit 562 | MIMAT0004762 | hsa-miR-486-3p; | Inf | 5.94E-06 |
| FaDu | MIMAT0000253 | hsa-miR-10a;hsa-miR-10a-5p; | 2.974848788 | 5.98E-06 |
| Cal 27 | MIMAT0004614 | hsa-miR-193a-5p; | -4.880989184 | 6.18E-06 |
| Cal 27 | MIMAT0000064 | hsa-let-7c;hsa-let-7c-5p; | -2.030255267 | 7.82E-06 |
| Detroit 562 | MIMAT0029782_1 |  | -5.605152374 | 8.96E-06 |
| H413 | MIMAT0000064 | hsa-let-7c;hsa-let-7c-5p; | -2.973234232 | 9.12E-06 |
| Detroit 562 | MIMAT0001536 | hsa-miR-429; | 3.699823069 | 1.26E-05 |
| FaDu | MIMAT0000257_1 |  | -2.927868866 | 1.35E-05 |
| Detroit 562 | MIMAT0000281 | hsa-miR-224;hsa-miR-224-5p; | 1.701936446 | 1.77E-05 |
| Detroit 562 | MIMAT0000419 | hsa-miR-27b;hsa-miR-27b-3p; | 1.499965997 | 1.78E-05 |
| Detroit 562 | MIMAT0004977 | hsa-miR-934; | Inf | 1.87E-05 |
| Detroit 562 | MIMAT0004954 | hsa-miR-543; | -5.272940812 | 2.02E-05 |
| Cal 27 | MIMAT0000510 | hsa-miR-320;hsa-miR-320a; | -1.647072219 | 2.10E-05 |
| Cal 27 | MIMAT0000440 | hsa-miR-191;hsa-miR-191-5p; | 1.511135605 | 2.37E-05 |
| FaDu | MIMAT0000436 | hsa-miR-144;hsa-miR-144-3p; | Inf | 2.62E-05 |
| H413 | MIMAT0000080_1 |  | 1.582749264 | 2.75E-05 |
| H413 | MIMAT0019208 | hsa-miR-3074-5p; | 1.582749264 | 2.75E-05 |
| Cal 27 | MIMAT0000088 | hsa-miR-30a-3p;hsa-miR-30a*;hsa-miR-30a-3p; | Inf | 3.43E-05 |
| Cal 27 | MIMAT0000083 | hsa-miR-26b;hsa-miR-26b-5p; | 3.49965447 | 6.51E-05 |
| Cal 27 | MIMAT0000078 | hsa-miR-23a;hsa-miR-23a-3p; | 2.183389066 | 7.08E-05 |
| FaDu | MIMAT0000099_1 |  | 2.649197423 | 7.46E-05 |
| FaDu | MIMAT0004592 | hsa-miR-125b-1*;hsa-miR-125b-1-3p; | -5.362993816 | 7.65E-05 |
| FaDu | MIMAT0004955 | hsa-miR-374b;hsa-miR-374b-5p; | 4.499331164 | 9.14E-05 |
| FaDu | MIMAT0022735 | hsa-miR-374c-3p; | 4.499331164 | 9.14E-05 |
| Detroit 562 | MIMAT0000088 | hsa-miR-30a-3p;hsa-miR-30a*;hsa-miR-30a-3p; | Inf | 0.000110566 |
| FaDu | MIMAT0029782_1 |  | -3.767159644 | 0.000121024 |
| Detroit 562 | MIMAT0000062 | hsa-let-7a;hsa-let-7a-5p; | -1.764530229 | 0.000145729 |
| Detroit 562 | MIMAT0015043 | hsa-miR-3168; | -6.541746742 | 0.000146415 |
| Detroit 562 | MIMAT0000083 | hsa-miR-26b;hsa-miR-26b-5p; | 3.466377411 | 0.000147143 |
| Detroit 562 | MIMAT0000732 | hsa-miR-422b;hsa-miR-378;hsa-miR-378a-3p; | 1.418112084 | 0.000151814 |
| Detroit 562 | MIMAT0023712 | hsa-miR-6087; | -7.346461521 | 0.00017182 |
| Detroit 562 | MIMAT0000280 | hsa-miR-223;hsa-miR-223-3p; | Inf | 0.00017601 |
| Detroit 562 | MIMAT0001635 | hsa-miR-452;hsa-miR-452-5p; | 3.316779136 | 0.000225323 |
| H413 | MIMAT0000435 | hsa-miR-143;hsa-miR-143-3p; | Inf | 0.000232746 |
| Detroit 562 | MIMAT0000070 | hsa-miR-17-5p;hsa-miR-17;hsa-miR-17-5p; | 2.319880154 | 0.000242004 |
| Detroit 562 | MIMAT0000279 | hsa-miR-222;hsa-miR-222-3p; | -1.112329504 | 0.000248213 |
| Detroit 562 | MIMAT0000440 | hsa-miR-191;hsa-miR-191-5p; | 1.232134794 | 0.000248286 |
| Detroit 562 | MIMAT0000080_1 |  | 1.187886196 | 0.000315772 |
| Detroit 562 | MIMAT0019208 | hsa-miR-3074-5p; | 1.187886196 | 0.000315772 |
| Detroit 562 | MIMAT0004955 | hsa-miR-374b;hsa-miR-374b-5p; | 4.385512916 | 0.000364878 |
| Detroit 562 | MIMAT0022735 | hsa-miR-374c-3p; | 4.385512916 | 0.000364878 |
| Cal 27 | MIMAT0000449 | hsa-miR-146;hsa-miR-146a;hsa-miR-146a-5p; | 4.014524128 | 0.000390056 |
| H413 | MIMAT0000278 | hsa-miR-221;hsa-miR-221-3p; | 1.582432072 | 0.000415901 |
| H413 | MIMAT0000256_1 |  | 1.594434667 | 0.000486745 |
| H413 | MIMAT0001536 | hsa-miR-429; | 3.583298046 | 0.000486745 |
| FaDu | MIMAT0000682 | hsa-miR-200a;hsa-miR-200a-3p; | 2.114505582 | 0.000495239 |
| Detroit 562 | MIMAT0000420 | hsa-miR-30b;hsa-miR-30b-5p; | 4.776467098 | 0.000501474 |
| Cal 27 | MIMAT0029782_1 |  | -3.362486937 | 0.000509396 |
| H413 | MIMAT0004614 | hsa-miR-193a-5p; | -4.498578902 | 0.000603218 |
| H413 | MIMAT0002819 | hsa-miR-193b;hsa-miR-193b-3p; | 4.229882815 | 0.000673902 |
| Detroit 562 | MIMAT0000069_1 |  | 3.497182072 | 0.000756646 |
| H413 | MIMAT0000098 | hsa-miR-100;hsa-miR-100-5p; | -2.88170498 | 0.000900481 |
| H413 | MIMAT0029782_1 |  | -5.732881301 | 0.000900481 |
| FaDu | MIMAT0000078 | hsa-miR-23a;hsa-miR-23a-3p; | 1.752895993 | 0.000906332 |
| Cal 27 | MIMAT0000278 | hsa-miR-221;hsa-miR-221-3p; | 1.279997217 | 0.000928731 |
| FaDu | MIMAT0000063 | hsa-let-7b;hsa-let-7b-5p; | -1.215516426 | 0.000988213 |
| Cal 27 | MIMAT0000062 | hsa-let-7a;hsa-let-7a-5p; | -1.583725615 | 0.001025581 |
| Cal 27 | MIMAT0019045 | hsa-miR-4508; | -Inf | 0.001055677 |
| FaDu | MIMAT0000510 | hsa-miR-320;hsa-miR-320a; | -1.396061319 | 0.001086316 |
| FaDu | MIMAT0000062_2 |  | -1.323530761 | 0.001110884 |
| Cal 27 | MIMAT0004792 | hsa-miR-92b*;hsa-miR-92b-5p; | -4.005722216 | 0.001306436 |
| Cal 27 | MIMAT0030019 | hsa-miR-7704; | -2.25375675 | 0.001306436 |
| Cal 27 | MIMAT0004592 | hsa-miR-125b-1*;hsa-miR-125b-1-3p; | -4.262241979 | 0.001329095 |
| Cal 27 | MIMAT0000732 | hsa-miR-422b;hsa-miR-378;hsa-miR-378a-3p; | 1.37478576 | 0.001338492 |
| H413 | MIMAT0000062_2 |  | -1.568272179 | 0.001428147 |
| Detroit 562 | MIMAT0000689 | hsa-miR-99b;hsa-miR-99b-5p; | -1.642522464 | 0.001583614 |
| FaDu | MIMAT0000692 | hsa-miR-30e-5p;hsa-miR-30e;hsa-miR-30e-5p; | 2.423556212 | 0.00158845 |
| H413 | MIMAT0015043 | hsa-miR-3168; | -6.201892516 | 0.001880992 |
| H413 | MIMAT0000080 | hsa-miR-24;hsa-miR-24-3p; | 1.63136203 | 0.001933814 |
| FaDu | MIMAT0000414 | hsa-let-7g;hsa-let-7g-5p; | 1.123252154 | 0.001943579 |
| H413 | MIMAT0000062_1 |  | -1.526584278 | 0.001989388 |
| FaDu | MIMAT0000075 | hsa-miR-20;hsa-miR-20a;hsa-miR-20a-5p; | 1.977056582 | 0.002072209 |
| FaDu | MIMAT0000226 | hsa-miR-196a;hsa-miR-196a-5p; | Inf | 0.002072209 |
| FaDu | MIMAT0000318 | hsa-miR-200b;hsa-miR-200b-3p; | 1.406841314 | 0.002214315 |
| Cal 27 | MIMAT0023712 | hsa-miR-6087; | -4.885390728 | 0.002263712 |
| Detroit 562 | MIMAT0000415 | hsa-let-7i;hsa-let-7i-5p; | 0.92534745 | 0.002342569 |
| FaDu | MIMAT0000069_1 |  | 3.362302642 | 0.002482294 |
| H413 | MIMAT0000226 | hsa-miR-196a;hsa-miR-196a-5p; | Inf | 0.002536404 |
| Detroit 562 | MIMAT0002819 | hsa-miR-193b;hsa-miR-193b-3p; | 3.786002974 | 0.002595001 |
| Cal 27 | MIMAT0000455 | hsa-miR-185;hsa-miR-185-5p; | 3.286075267 | 0.002647593 |
| FaDu | MIMAT0023712 | hsa-miR-6087; | -4.779083192 | 0.002745037 |
| FaDu | MIMAT0000062_1 |  | -1.222078347 | 0.002782856 |
| FaDu | MIMAT0002819 | hsa-miR-193b;hsa-miR-193b-3p; | 3.751534574 | 0.003035453 |
| Detroit 562 | MIMAT0000418 | hsa-miR-23b;hsa-miR-23b-3p; | 2.347442754 | 0.003141425 |
| FaDu | MIMAT0000082_1 |  | 1.201746579 | 0.003221394 |
| FaDu | MIMAT0000433 | hsa-miR-142-5p; | 5.210774203 | 0.00335157 |
| Detroit 562 | MIMAT0000510 | hsa-miR-320;hsa-miR-320a; | -1.363175444 | 0.003417791 |
| Cal 27 | MIMAT0000066 | hsa-let-7e;hsa-let-7e-5p; | -1.950667342 | 0.003525612 |
| FaDu | MIMAT0000232 | hsa-miR-199a*;hsa-miR-199a-3p; | Inf | 0.004025329 |
| FaDu | MIMAT0003393 | hsa-miR-425-5p;hsa-miR-425;hsa-miR-425-5p; | 2.227068933 | 0.004176459 |
| FaDu | MIMAT0000070 | hsa-miR-17-5p;hsa-miR-17;hsa-miR-17-5p; | 1.956932314 | 0.004310953 |
| H413 | MIMAT0000244 | hsa-miR-30c;hsa-miR-30c-5p; | 2.366838887 | 0.00456114 |
| FaDu | MIMAT0004792 | hsa-miR-92b*;hsa-miR-92b-5p; | -3.678886473 | 0.004941559 |
| Detroit 562 | MIMAT0000256 | hsa-miR-181a;hsa-miR-181a-5p; | 1.869228819 | 0.004975628 |
| Detroit 562 | MIMAT0004792 | hsa-miR-92b*;hsa-miR-92b-5p; | -4.076758266 | 0.005134638 |
| FaDu | MIMAT0000264 | hsa-miR-203;hsa-miR-203a; | 1.475968729 | 0.005268074 |
| FaDu | MIMAT0019813 | hsa-miR-3545-5p;hsa-miR-203b-5p; | 1.475968729 | 0.005268074 |
| Cal 27 | MIMAT0000456 | hsa-miR-186;hsa-miR-186-5p; | 2.8057532 | 0.005333016 |
| Cal 27 | MIMAT0000222 | hsa-miR-192;hsa-miR-192-5p; | 3.125007114 | 0.005390489 |
| FaDu | MIMAT0000082 | hsa-miR-26a;hsa-miR-26a-5p; | 1.823361959 | 0.005683665 |
| Cal 27 | MIMAT0000082_1 |  | 1.119134001 | 0.005827712 |
| Detroit 562 | MIMAT0019045 | hsa-miR-4508; | -7.600355222 | 0.006162603 |
| FaDu | MIMAT0005883 | hsa-miR-1293; | -4.826137836 | 0.006479189 |
| FaDu | MIMAT0015043 | hsa-miR-3168; | -4.074691769 | 0.007057144 |
| Detroit 562 | MIMAT0000680 | hsa-miR-106b;hsa-miR-106b-5p; | 2.428600542 | 0.007406139 |
| FaDu | MIMAT0004954 | hsa-miR-543; | -2.959444824 | 0.007503344 |
| FaDu | MIMAT0000444 | hsa-miR-126*;hsa-miR-126-5p; | 5.051457584 | 0.008063872 |
| FaDu | MIMAT0005951 | hsa-miR-1307;hsa-miR-1307-3p; | -1.170025529 | 0.00857994 |
| Cal 27 | MIMAT0002819 | hsa-miR-193b;hsa-miR-193b-3p; | 3.577370944 | 0.009070591 |
| Detroit 562 | MIMAT0000423_1 |  | -1.573151341 | 0.00909865 |
| Cal 27 | MIMAT0000253 | hsa-miR-10a;hsa-miR-10a-5p; | 2.270914676 | 0.00935807 |
| FaDu | MIMAT0000064 | hsa-let-7c;hsa-let-7c-5p; | -1.392894056 | 0.00948649 |
| Cal 27 | MIMAT0000259 | hsa-miR-182;hsa-miR-182-5p; | 1.933391647 | 0.010018577 |
| FaDu | MIMAT0000680 | hsa-miR-106b;hsa-miR-106b-5p; | 2.385238808 | 0.010564973 |
| FaDu | MIMAT0000686 | hsa-miR-34c;hsa-miR-34c-5p; | -Inf | 0.010564973 |
| H413 | MIMAT0030019 | hsa-miR-7704; | -3.520805065 | 0.010781603 |
| Cal 27 | MIMAT0000067 | hsa-let-7f;hsa-let-7f-5p; | 0.88694594 | 0.011012426 |
| H413 | MIMAT0004792 | hsa-miR-92b*;hsa-miR-92b-5p; | -4.911614039 | 0.011396236 |
| H413 | MIMAT0000259 | hsa-miR-182;hsa-miR-182-5p; | 2.112856442 | 0.011874047 |
| FaDu | MIMAT0004563 | hsa-miR-199b-3p; | Inf | 0.012145184 |
| FaDu | MIMAT0000259 | hsa-miR-182;hsa-miR-182-5p; | 1.881730658 | 0.012628459 |
| FaDu | MIMAT0000244_1 |  | 3.266845348 | 0.01263823 |
| Detroit 562 | MIMAT0000066 | hsa-let-7e;hsa-let-7e-5p; | -1.843708878 | 0.012906448 |
| Cal 27 | MIMAT0000420 | hsa-miR-30b;hsa-miR-30b-5p; | 4.337665199 | 0.013582202 |
| FaDu | MIMAT0000434 | hsa-miR-142-3p; | Inf | 0.014631129 |
| FaDu | MIMAT0004703 | hsa-miR-335*;hsa-miR-335-3p; | Inf | 0.015202757 |
| Detroit 562 | MIMAT0019032 | hsa-miR-4497; | -6.765458145 | 0.015932106 |
| Detroit 562 | MIMAT0004703 | hsa-miR-335*;hsa-miR-335-3p; | Inf | 0.018475573 |
| FaDu | MIMAT0000455 | hsa-miR-185;hsa-miR-185-5p; | 2.929129914 | 0.018581308 |
| Cal 27 | MIMAT0000075 | hsa-miR-20;hsa-miR-20a;hsa-miR-20a-5p; | 1.629814053 | 0.019350899 |
| Detroit 562 | MIMAT0000259 | hsa-miR-182;hsa-miR-182-5p; | 1.791610582 | 0.019685808 |
| Detroit 562 | MIMAT0004926 | hsa-miR-708;hsa-miR-708-5p; | -7.169312487 | 0.019685808 |
| H413 | MIMAT0000423_1 |  | -2.017934738 | 0.019977762 |
| H413 | MIMAT0004954 | hsa-miR-543; | -4.178929056 | 0.020129452 |
| FaDu | MIMAT0003322 | hsa-miR-652;hsa-miR-652-3p; | 4.842835676 | 0.020830682 |
| Cal 27 | MIMAT0001620 | hsa-miR-200a*;hsa-miR-200a-5p; | 4.534849669 | 0.020833173 |
| FaDu | MIMAT0000764 | hsa-miR-339;hsa-miR-339-5p; | 3.162427794 | 0.021192873 |
| Cal 27 | MIMAT0000764 | hsa-miR-339;hsa-miR-339-5p; | 3.212395481 | 0.021380541 |
| FaDu | MIMAT0002816 | hsa-miR-494;hsa-miR-494-3p; | Inf | 0.02178028 |
| Detroit 562 | MIMAT0003322 | hsa-miR-652;hsa-miR-652-3p; | 4.966326675 | 0.023345131 |
| Detroit 562 | MIMAT0004672 | hsa-miR-106b*;hsa-miR-106b-3p; | 2.265794758 | 0.024431263 |
| FaDu | MIMAT0000232_1 |  | Inf | 0.024857419 |
| Detroit 562 | MIMAT0000451 | hsa-miR-150;hsa-miR-150-5p; | Inf | 0.025061076 |
| Cal 27 | MIMAT0004926 | hsa-miR-708;hsa-miR-708-5p; | -6.746728014 | 0.026031789 |
| Detroit 562 | MIMAT0000447 | hsa-miR-134;hsa-miR-134-5p; | -5.407026351 | 0.026695986 |
| H413 | MIMAT0000757 | hsa-miR-151;hsa-miR-151-3p;hsa-miR-151a-3p; | 1.62786582 | 0.027203204 |
| H413 | MIMAT0023712 | hsa-miR-6087; | -5.294242198 | 0.027203204 |
| Cal 27 | MIMAT0000250 | hsa-miR-139;hsa-miR-139-5p; | 3.627136526 | 0.027253229 |
| H413 | MIMAT0004748 | hsa-miR-423-5p; | -1.686206294 | 0.028169198 |
| H413 | MIMAT0022731 | hsa-miR-3184-3p; | -1.686206294 | 0.028169198 |
| FaDu | MIMAT0002174 | hsa-miR-484; | 2.655101652 | 0.028329076 |
| Cal 27 | MIMAT0000434 | hsa-miR-142-3p; | Inf | 0.028661486 |
| Detroit 562 | MIMAT0000082_1 |  | 0.882911989 | 0.030096135 |
| Detroit 562 | MIMAT0000252 | hsa-miR-7;hsa-miR-7-5p; | 1.483402405 | 0.030096135 |
| Detroit 562 | MIMAT0004748 | hsa-miR-423-5p; | -1.129126964 | 0.030096135 |
| Detroit 562 | MIMAT0022731 | hsa-miR-3184-3p; | -1.129126964 | 0.030096135 |
| Detroit 562 | MIMAT0022741 | hsa-miR-3529-3p; | 1.483402405 | 0.030096135 |
| FaDu | MIMAT0004597 | hsa-miR-140-3p; | 1.739890863 | 0.030522197 |
| Cal 27 | MIMAT0003393 | hsa-miR-425-5p;hsa-miR-425;hsa-miR-425-5p; | 1.976901922 | 0.030527582 |
| FaDu | MIMAT0004600 | hsa-miR-144*;hsa-miR-144-5p; | Inf | 0.031642687 |
| Detroit 562 | MIMAT0000082 | hsa-miR-26a;hsa-miR-26a-5p; | 1.593589026 | 0.033056438 |
| FaDu | MIMAT0000689 | hsa-miR-99b;hsa-miR-99b-5p; | -1.142003512 | 0.033301152 |
| H413 | MIMAT0000097 | hsa-miR-99a;hsa-miR-99a-5p; | -2.099047017 | 0.03352051 |
| FaDu | MIMAT0000101 | hsa-miR-103;hsa-miR-103a;hsa-miR-103a-3p; | 1.346480084 | 0.033563048 |
| FaDu | MIMAT0007402 | hsa-miR-103-as;hsa-miR-103b; | 1.346480084 | 0.033563048 |
| Detroit 562 | MIMAT0000244_1 |  | 3.150224308 | 0.034324305 |
| Detroit 562 | MIMAT0002874 | hsa-miR-503;hsa-miR-503-5p; | -2.149436132 | 0.034595903 |
| Detroit 562 | MIMAT0000252_1 |  | 1.558513909 | 0.035438993 |
| Detroit 562 | MIMAT0000253 | hsa-miR-10a;hsa-miR-10a-5p; | 2.100965389 | 0.035438993 |
| Detroit 562 | MIMAT0005883 | hsa-miR-1293; | -4.161912281 | 0.035696823 |
| Cal 27 | MIMAT0000418 | hsa-miR-23b;hsa-miR-23b-3p; | 2.028912917 | 0.037699882 |
| H413 | MIMAT0000077 | hsa-miR-22;hsa-miR-22-3p; | 0.921500891 | 0.043066679 |
| Cal 27 | MIMAT0003329 | hsa-miR-411;hsa-miR-411-5p; | 2.260761919 | 0.043073744 |
| FaDu | MIMAT0000257 | hsa-miR-181b;hsa-miR-181b-5p; | -2.887220188 | 0.043469585 |
| FaDu | MIMAT0019045 | hsa-miR-4508; | -5.609577674 | 0.044373395 |
| FaDu | MIMAT0001341 | hsa-miR-424;hsa-miR-424-5p; | -3.670611761 | 0.045893569 |
| Detroit 562 | MIMAT0000254 | hsa-miR-10b;hsa-miR-10b-5p; | 4.782104734 | 0.047594542 |
| FaDu | MIMAT0000753 | hsa-miR-342;hsa-miR-342-3p; | 4.629562751 | 0.048571988 |
| Detroit 562 | MIMAT0000075 | hsa-miR-20;hsa-miR-20a;hsa-miR-20a-5p; | 1.796603733 | 0.049599778 |
| FaDu | MIMAT0019032 | hsa-miR-4497; | -4.661275873 | 0.049966016 |
| Cal 27 | MIMAT0000414 | hsa-let-7g;hsa-let-7g-5p; | 0.863201404 | 0.050447037 |
| Cal 27 | MIMAT0005951 | hsa-miR-1307;hsa-miR-1307-3p; | -0.912895568 | 0.051196752 |
| Cal 27 | MIMAT0018076 | hsa-miR-3656; | -Inf | 0.051510076 |
| Cal 27 | MIMAT0004955 | hsa-miR-374b;hsa-miR-374b-5p; | 3.541943363 | 0.051676933 |
| Cal 27 | MIMAT0022735 | hsa-miR-374c-3p; | 3.541943363 | 0.051676933 |
| Cal 27 | MIMAT0000082 | hsa-miR-26a;hsa-miR-26a-5p; | 1.524308238 | 0.058047532 |
| Detroit 562 | MIMAT0000097 | hsa-miR-99a;hsa-miR-99a-5p; | -1.90687226 | 0.05851085 |
| Cal 27 | MIMAT0002816 | hsa-miR-494;hsa-miR-494-3p; | Inf | 0.059015519 |
| Cal 27 | MIMAT0003322 | hsa-miR-652;hsa-miR-652-3p; | 4.614011916 | 0.059318994 |
| Cal 27 | MIMAT0004954 | hsa-miR-543; | -2.33273306 | 0.059318994 |
| Cal 27 | MIMAT0015043 | hsa-miR-3168; | -3.332600801 | 0.059318994 |
| Detroit 562 | MIMAT0000456 | hsa-miR-186;hsa-miR-186-5p; | 2.379776262 | 0.060396655 |
| FaDu | MIMAT0005882 | hsa-miR-548k; | Inf | 0.060644741 |
| Cal 27 | MIMAT0000080_1 |  | 0.749109869 | 0.062869648 |
| Cal 27 | MIMAT0019208 | hsa-miR-3074-5p; | 0.749109869 | 0.062869648 |
| Cal 27 | MIMAT0000257_1 |  | -1.377974648 | 0.062994988 |
| FaDu | MIMAT0000646 | hsa-miR-155;hsa-miR-155-5p; | 2.383068482 | 0.06333873 |
| Cal 27 | MIMAT0001341 | hsa-miR-424;hsa-miR-424-5p; | -3.562434075 | 0.06372313 |
| Cal 27 | MIMAT0000244_1 |  | 2.963411437 | 0.064996984 |
| Detroit 562 | MIMAT0000101 | hsa-miR-103;hsa-miR-103a;hsa-miR-103a-3p; | 1.214690912 | 0.065751699 |
| Detroit 562 | MIMAT0007402 | hsa-miR-103-as;hsa-miR-103b; | 1.214690912 | 0.065751699 |
| FaDu | MIMAT0000250 | hsa-miR-139;hsa-miR-139-5p; | 3.395604633 | 0.065964207 |
| FaDu | MIMAT0004926 | hsa-miR-708;hsa-miR-708-5p; | -5.027593521 | 0.065964207 |
| H413 | MIMAT0000438 | hsa-miR-152;hsa-miR-152-3p; | 3.157752391 | 0.067625509 |
| H413 | MIMAT0000082 | hsa-miR-26a;hsa-miR-26a-5p; | 1.650380458 | 0.067675461 |
| FaDu | MIMAT0003329 | hsa-miR-411;hsa-miR-411-5p; | 2.311344915 | 0.070674862 |
| Cal 27 | MIMAT0000076 | hsa-miR-21;hsa-miR-21-5p; | -0.76253914 | 0.074388381 |
| FaDu | MIMAT0000069 | hsa-miR-16;hsa-miR-16-5p; | 3.534307164 | 0.077138363 |
| Cal 27 | MIMAT0000069_1 |  | 2.860966641 | 0.081231567 |
| Cal 27 | MIMAT0000099_1 |  | 1.783089659 | 0.081231567 |
| Cal 27 | MIMAT0000232 | hsa-miR-199a*;hsa-miR-199a-3p; | Inf | 0.081231567 |
| Cal 27 | MIMAT0000279 | hsa-miR-222;hsa-miR-222-3p; | -0.772226988 | 0.081231567 |
| Cal 27 | MIMAT0000433 | hsa-miR-142-5p; | 4.508113659 | 0.081231567 |
| Cal 27 | MIMAT0000444 | hsa-miR-126*;hsa-miR-126-5p; | 4.505346718 | 0.081231567 |
| Cal 27 | MIMAT0004597 | hsa-miR-140-3p; | 1.582193251 | 0.081231567 |
| H413 | MIMAT0000087 | hsa-miR-30a-5p;hsa-miR-30a;hsa-miR-30a-5p; | 1.246806554 | 0.084402977 |
| Cal 27 | MIMAT0004702 | hsa-miR-339-3p; | 4.487833628 | 0.086917922 |
| Detroit 562 | MIMAT0004592 | hsa-miR-125b-1*;hsa-miR-125b-1-3p; | -3.097540986 | 0.087459433 |
| FaDu | MIMAT0000067 | hsa-let-7f;hsa-let-7f-5p; | 0.718635025 | 0.089405927 |
| Detroit 562 | MIMAT0004945 | hsa-miR-744;hsa-miR-744-5p; | 1.97431701 | 0.095893317 |
| FaDu | MIMAT0004494 | hsa-miR-21*;hsa-miR-21-3p; | 4.050209465 | 0.099196326 |
| Cal 27 | MIMAT0019047 | hsa-miR-4510; | -2.900995617 | 0.099896478 |

**Supplementary Table 2: KEGG pathways that were enriched among experimentally supported (TarBase) targets of miRNA that were differentially secreted by all 4 HNSCC cell lines relative to primary non-pathologic oral epithelial control cells**

| **KEGG pathway** | **p-value** | **#genes** | **#miRNAs** |
| --- | --- | --- | --- |
| MicroRNAs in cancer | 1.48E-69 | 132 | 24 |
| Proteoglycans in cancer | 1.18E-14 | 146 | 23 |
| Protein processing in endoplasmic reticulum | 1.71E-11 | 133 | 23 |
| Cell cycle | 1.87E-11 | 97 | 21 |
| Adherens junction | 7.96E-09 | 61 | 21 |
| TGF-beta signaling pathway | 4.88E-08 | 63 | 21 |
| Lysine degradation | 9.49E-08 | 38 | 20 |
| Hippo signaling pathway | 9.49E-08 | 104 | 23 |
| Spliceosome | 3.49E-07 | 100 | 22 |
| Viral carcinogenesis | 4.48E-07 | 146 | 23 |
| Pathways in cancer | 4.48E-07 | 260 | 23 |
| Colorectal cancer | 7.65E-07 | 52 | 23 |
| RNA transport | 1.59E-06 | 124 | 22 |
| Thyroid hormone signaling pathway | 3.80E-06 | 87 | 23 |
| p53 signaling pathway | 6.77E-06 | 57 | 22 |
| Renal cell carcinoma | 1.50E-05 | 54 | 20 |
| Signaling pathways regulating pluripotency of stem cells | 1.81E-05 | 99 | 20 |
| Hepatitis B | 1.97E-05 | 99 | 23 |
| Endocytosis | 2.08E-05 | 142 | 23 |
| Shigellosis | 2.84E-05 | 50 | 22 |
| Chronic myeloid leukemia | 3.86E-05 | 57 | 22 |
| Ribosome | 4.41E-05 | 99 | 22 |
| Prostate cancer | 4.41E-05 | 68 | 23 |
| Pancreatic cancer | 4.62E-05 | 51 | 22 |
| FoxO signaling pathway | 4.62E-05 | 98 | 23 |
| Ubiquitin mediated proteolysis | 6.55E-05 | 102 | 24 |
| Insulin signaling pathway | 9.19E-05 | 101 | 22 |
| Bacterial invasion of epithelial cells | 9.19E-05 | 56 | 22 |
| Transcriptional misregulation in cancer | 0.000104138 | 116 | 22 |
| Endometrial cancer | 0.000152968 | 42 | 23 |
| Prion diseases | 0.000191391 | 20 | 18 |
| Non-small cell lung cancer | 0.000194925 | 43 | 22 |
| Acute myeloid leukemia | 0.000296113 | 45 | 20 |
| AMPK signaling pathway | 0.000296113 | 88 | 22 |
| Neurotrophin signaling pathway | 0.000334322 | 85 | 23 |
| Steroid biosynthesis | 0.000370824 | 15 | 15 |
| mRNA surveillance pathway | 0.000370824 | 68 | 22 |
| mTOR signaling pathway | 0.000393802 | 48 | 22 |
| Small cell lung cancer | 0.000411456 | 64 | 23 |
| Oocyte meiosis | 0.000535838 | 75 | 20 |
| Glioma | 0.000606111 | 46 | 22 |
| Fatty acid metabolism | 0.000756837 | 30 | 22 |
| Regulation of actin cytoskeleton | 0.000756837 | 136 | 23 |
| Central carbon metabolism in cancer | 0.000789635 | 49 | 22 |
| Thyroid cancer | 0.000816534 | 24 | 19 |
| Sphingolipid signaling pathway | 0.000816534 | 81 | 22 |
| N-Glycan biosynthesis | 0.001266432 | 35 | 18 |
| DNA replication | 0.001883442 | 29 | 16 |
| Focal adhesion | 0.002310169 | 135 | 23 |
| Epstein-Barr virus infection | 0.003631143 | 134 | 23 |
| Bladder cancer | 0.005733595 | 31 | 20 |
| ErbB signaling pathway | 0.005733595 | 59 | 22 |
| Prolactin signaling pathway | 0.005733595 | 50 | 22 |
| HTLV-I infection | 0.005733595 | 165 | 24 |
| HIF-1 signaling pathway | 0.005769851 | 73 | 22 |
| Other types of O-glycan biosynthesis | 0.006980332 | 18 | 15 |
| Fc gamma R-mediated phagocytosis | 0.007900082 | 63 | 23 |
| Choline metabolism in cancer | 0.009139862 | 70 | 21 |
| Glycosaminoglycan biosynthesis - keratan sulfate | 0.010675386 | 12 | 15 |
| Fatty acid elongation | 0.010949871 | 14 | 13 |
| SNARE interactions in vesicular transport | 0.010959962 | 25 | 18 |
| Huntington's disease | 0.010959962 | 116 | 23 |
| TNF signaling pathway | 0.014318708 | 74 | 23 |
| MAPK signaling pathway | 0.015932552 | 155 | 24 |
| Progesterone-mediated oocyte maturation | 0.022805816 | 60 | 21 |
| Non-alcoholic fatty liver disease (NAFLD) | 0.023245688 | 97 | 23 |
| Proteasome | 0.024906945 | 34 | 18 |
| Estrogen signaling pathway | 0.024916852 | 65 | 22 |
| Vitamin B6 metabolism | 0.02731842 | 5 | 8 |
| Fatty acid biosynthesis | 0.034253994 | 7 | 17 |
| Salmonella infection | 0.034253994 | 57 | 23 |
| Mismatch repair | 0.035851741 | 18 | 17 |
| Basal transcription factors | 0.035851741 | 33 | 19 |
| Wnt signaling pathway | 0.036827672 | 84 | 22 |
| RNA degradation | 0.037028955 | 55 | 22 |
| Pathogenic Escherichia coli infection | 0.037580106 | 39 | 22 |
| Base excision repair | 0.037966678 | 24 | 15 |
| Melanoma | 0.041591116 | 47 | 22 |

**Supplementary Table 3: KEGG pathways that were enriched among predicted (MicroT-CDS) targets of miRNA that were differentially secreted by all 4 HNSCC cell lines relative to primary non-pathologic oral epithelial control cells**

| **KEGG pathway** | **p-value** | **#genes** | **#miRNAs** |
| --- | --- | --- | --- |
| Proteoglycans in cancer | 2.78E-10 | 119 | 25 |
| ECM-receptor interaction | 1.10E-06 | 44 | 22 |
| Axon guidance | 1.10E-06 | 77 | 24 |
| ErbB signaling pathway | 1.10E-06 | 58 | 25 |
| GABAergic synapse | 2.37E-05 | 47 | 22 |
| Glioma | 3.70E-05 | 40 | 24 |
| Regulation of actin cytoskeleton | 4.15E-05 | 117 | 26 |
| Neurotrophin signaling pathway | 0.000154349 | 75 | 27 |
| Hippo signaling pathway | 0.000269445 | 74 | 23 |
| Morphine addiction | 0.000269445 | 48 | 25 |
| Prion diseases | 0.000373859 | 11 | 13 |
| Bacterial invasion of epithelial cells | 0.000377742 | 45 | 22 |
| Focal adhesion | 0.000377742 | 113 | 28 |
| PI3K-Akt signaling pathway | 0.000500588 | 174 | 27 |
| Thyroid hormone signaling pathway | 0.000525043 | 63 | 23 |
| MAPK signaling pathway | 0.000525043 | 134 | 27 |
| Estrogen signaling pathway | 0.000633635 | 52 | 24 |
| Renal cell carcinoma | 0.000633635 | 43 | 24 |
| Ras signaling pathway | 0.000633635 | 114 | 26 |
| Adrenergic signaling in cardiomyocytes | 0.000830267 | 76 | 25 |
| Amphetamine addiction | 0.001099731 | 37 | 18 |
| TGF-beta signaling pathway | 0.001154449 | 47 | 25 |
| Pathways in cancer | 0.00119743 | 198 | 28 |
| Rap1 signaling pathway | 0.001401955 | 109 | 26 |
| Adherens junction | 0.001841463 | 47 | 22 |
| Mucin type O-Glycan biosynthesis | 0.001845518 | 16 | 15 |
| FoxO signaling pathway | 0.001845518 | 75 | 25 |
| Chronic myeloid leukemia | 0.002489848 | 43 | 22 |
| Long-term potentiation | 0.002547747 | 42 | 23 |
| Prostate cancer | 0.003088883 | 52 | 24 |
| mTOR signaling pathway | 0.003772273 | 38 | 24 |
| cGMP-PKG signaling pathway | 0.004056174 | 87 | 26 |
| Oxytocin signaling pathway | 0.00411322 | 84 | 25 |
| Melanoma | 0.004826921 | 42 | 22 |
| Nicotine addiction | 0.005395601 | 23 | 20 |
| Retrograde endocannabinoid signaling | 0.005395601 | 56 | 24 |
| Long-term depression | 0.006408185 | 36 | 21 |
| Glutamatergic synapse | 0.006517879 | 60 | 23 |
| Dopaminergic synapse | 0.006517879 | 72 | 24 |
| Sphingolipid signaling pathway | 0.007517878 | 61 | 22 |
| AMPK signaling pathway | 0.008807284 | 68 | 26 |
| Choline metabolism in cancer | 0.009256588 | 58 | 24 |
| T cell receptor signaling pathway | 0.009432366 | 57 | 23 |
| Glycosaminoglycan biosynthesis - keratan sulfate | 0.010448364 | 9 | 13 |
| Non-small cell lung cancer | 0.012918814 | 34 | 24 |
| Gap junction | 0.024643299 | 45 | 21 |
| Viral carcinogenesis | 0.026037726 | 82 | 24 |
| Wnt signaling pathway | 0.02735352 | 71 | 22 |
| Tight junction | 0.028231927 | 71 | 24 |
| Cocaine addiction | 0.02943739 | 26 | 17 |
| Ubiquitin mediated proteolysis | 0.02943739 | 71 | 24 |
| N-Glycan biosynthesis | 0.029565653 | 23 | 16 |
| Transcriptional misregulation in cancer | 0.029565653 | 82 | 25 |
| Colorectal cancer | 0.029667054 | 36 | 20 |

**Supplementary Table 4: GO categories that were enriched among experimentally supported (TarBase) targets of miRNA that were differentially secreted by all 4 HNSCC cell lines relative to primary non-pathologic oral epithelial control cells**

| **GO Term** | **Enrichment p-value** | **# Genes Targeted** | **miRNAs involved** |
| --- | --- | --- | --- |
| cellular nitrogen compound metabolic process | < 1.00E-325 | 2909 | 24 |
| biosynthetic process | 1.53E-234 | 2374 | 24 |
| gene expression | 5.11E-214 | 537 | 24 |
| symbiosis, encompassing mutualism through parasitism | 2.29E-154 | 458 | 24 |
| viral process | 7.32E-151 | 415 | 24 |
| cellular protein modification process | 7.15E-144 | 1395 | 24 |
| small molecule metabolic process | 7.75E-137 | 1383 | 24 |
| catabolic process | 6.34E-127 | 1197 | 24 |
| mitotic cell cycle | 6.41E-107 | 329 | 24 |
| cellular protein metabolic process | 4.31E-105 | 360 | 24 |
| nucleobase-containing compound catabolic process | 7.33E-85 | 597 | 24 |
| membrane organization | 2.11E-77 | 417 | 24 |
| biological_process | 4.47E-75 | 7693 | 24 |
| cellular component assembly | 1.19E-69 | 761 | 24 |
| mRNA metabolic process | 6.52E-69 | 186 | 24 |
| RNA metabolic process | 1.82E-64 | 206 | 24 |
| neurotrophin TRK receptor signaling pathway | 2.89E-62 | 186 | 24 |
| macromolecular complex assembly | 1.49E-60 | 538 | 24 |
| response to stress | 2.58E-59 | 1186 | 24 |
| cell death | 2.26E-45 | 530 | 23 |
| post-translational protein modification | 1.37E-44 | 129 | 22 |
| DNA metabolic process | 2.39E-44 | 466 | 24 |
| blood coagulation | 9.28E-42 | 270 | 23 |
| protein complex assembly | 3.37E-39 | 441 | 24 |
| Fc-epsilon receptor signaling pathway | 1.42E-37 | 110 | 24 |
| viral life cycle | 2.60E-35 | 94 | 23 |
| viral transcription | 9.21E-28 | 69 | 21 |
| cellular lipid metabolic process | 9.57E-28 | 102 | 23 |
| TRIF-dependent toll-like receptor signaling pathway | 5.92E-27 | 59 | 21 |
| generation of precursor metabolites and energy | 1.89E-26 | 206 | 24 |
| G2/M transition of mitotic cell cycle | 2.95E-23 | 110 | 23 |
| epidermal growth factor receptor signaling pathway | 4.90E-23 | 132 | 23 |
| toll-like receptor 10 signaling pathway | 1.08E-22 | 50 | 21 |
| MyD88-independent toll-like receptor signaling pathway | 1.25E-22 | 59 | 21 |
| toll-like receptor TLR1:TLR2 signaling pathway | 1.46E-22 | 52 | 21 |
| toll-like receptor TLR6:TLR2 signaling pathway | 1.46E-22 | 52 | 21 |
| transcription initiation from RNA polymerase II promoter | 1.24E-21 | 146 | 23 |
| protein targeting | 1.50E-21 | 185 | 23 |
| chromatin organization | 2.49E-21 | 96 | 20 |
| mitotic nuclear envelope disassembly | 1.13E-20 | 35 | 18 |
| regulation of ubiquitin-protein ligase activity involved in mitotic cell cycle | 9.31E-20 | 57 | 20 |
| activation of signaling protein activity involved in unfolded protein response | 1.06E-19 | 52 | 20 |
| translational termination | 1.43E-19 | 73 | 22 |
| Fc-gamma receptor signaling pathway involved in phagocytosis | 2.37E-19 | 52 | 22 |
| protein N-linked glycosylation via asparagine | 2.37E-19 | 73 | 22 |
| toll-like receptor 3 signaling pathway | 2.91E-19 | 60 | 21 |
| SRP-dependent cotranslational protein targeting to membrane | 2.98E-19 | 89 | 22 |
| toll-like receptor 5 signaling pathway | 1.40E-18 | 50 | 21 |
| nuclear-transcribed mRNA catabolic process, nonsense-mediated decay | 1.89E-18 | 97 | 22 |
| positive regulation of ubiquitin-protein ligase activity involved in mitotic cell cycle | 3.71E-18 | 54 | 19 |
| immune system process | 4.31E-18 | 734 | 24 |
| toll-like receptor 9 signaling pathway | 1.07E-17 | 52 | 21 |
| cellular component disassembly involved in execution phase of apoptosis | 1.21E-17 | 38 | 20 |
| platelet activation | 1.34E-17 | 116 | 23 |
| G1/S transition of mitotic cell cycle | 2.03E-17 | 123 | 23 |
| cell junction organization | 5.43E-16 | 98 | 22 |
| vitamin metabolic process | 6.75E-16 | 53 | 18 |
| water-soluble vitamin metabolic process | 8.95E-16 | 49 | 18 |
| negative regulation of ubiquitin-protein ligase activity involved in mitotic cell cycle | 3.40E-15 | 49 | 19 |
| toll-like receptor 4 signaling pathway | 3.60E-15 | 64 | 21 |
| anaphase-promoting complex-dependent proteasomal ubiquitin-dependent protein catabolic process | 4.35E-15 | 62 | 20 |
| nucleobase-containing small molecule metabolic process | 1.96E-14 | 47 | 21 |
| fibroblast growth factor receptor signaling pathway | 2.66E-14 | 112 | 23 |
| toll-like receptor 2 signaling pathway | 3.85E-14 | 52 | 21 |
| RNA splicing | 5.53E-14 | 193 | 23 |
| hexose transport | 7.23E-14 | 32 | 18 |
| stress-activated MAPK cascade | 7.92E-14 | 42 | 21 |
| ribonucleoprotein complex assembly | 2.19E-13 | 95 | 22 |
| toll-like receptor signaling pathway | 2.50E-13 | 69 | 22 |
| mRNA processing | 2.95E-13 | 292 | 24 |
| transcription, DNA-templated | 5.81E-13 | 1152 | 24 |
| cell junction assembly | 6.40E-13 | 44 | 20 |
| glycosaminoglycan metabolic process | 1.84E-12 | 62 | 21 |
| cell cycle | 2.61E-12 | 471 | 24 |
| termination of RNA polymerase II transcription | 7.10E-12 | 35 | 18 |
| regulation of glucose transport | 1.11E-11 | 27 | 18 |
| DNA damage response, signal transduction by p53 class mediator resulting in cell cycle arrest | 1.49E-11 | 51 | 20 |
| glycerophospholipid biosynthetic process | 1.49E-11 | 54 | 20 |
| cofactor metabolic process | 1.50E-11 | 133 | 22 |
| mRNA 3'-end processing | 3.42E-11 | 32 | 18 |
| phospholipid metabolic process | 7.32E-11 | 95 | 23 |
| intrinsic apoptotic signaling pathway | 1.71E-10 | 49 | 22 |
| regulation of cellular amino acid metabolic process | 2.02E-10 | 37 | 19 |
| post-Golgi vesicle-mediated transport | 3.55E-10 | 37 | 18 |
| extracellular matrix disassembly | 4.52E-10 | 61 | 24 |
| nuclear-transcribed mRNA catabolic process, deadenylation-dependent decay | 1.06E-09 | 42 | 21 |
| cellular metabolic process | 1.06E-09 | 94 | 22 |
| respiratory electron transport chain | 1.45E-09 | 64 | 17 |
| vesicle-mediated transport | 1.73E-09 | 475 | 24 |
| MyD88-dependent toll-like receptor signaling pathway | 4.31E-09 | 55 | 21 |
| nucleotide-binding domain, leucine rich repeat containing receptor signaling pathway | 5.22E-09 | 28 | 21 |
| tRNA metabolic process | 5.78E-09 | 97 | 18 |
| platelet degranulation | 8.94E-09 | 42 | 21 |
| nucleocytoplasmic transport | 9.45E-09 | 183 | 24 |
| insulin receptor signaling pathway | 9.60E-09 | 94 | 21 |
| leukocyte migration | 1.02E-08 | 65 | 22 |
| phosphatidylinositol biosynthetic process | 1.94E-08 | 42 | 22 |
| DNA strand elongation involved in DNA replication | 2.18E-08 | 24 | 16 |
| positive regulation of protein insertion into mitochondrial membrane involved in apoptotic signaling pathway | 3.71E-08 | 23 | 21 |
| phosphatidylinositol-mediated signaling | 5.59E-08 | 73 | 23 |
| transcription elongation from RNA polymerase II promoter | 7.45E-08 | 49 | 21 |
| energy reserve metabolic process | 9.85E-08 | 53 | 21 |
| innate immune response | 1.59E-07 | 316 | 24 |
| extracellular matrix organization | 2.08E-07 | 176 | 24 |
| antigen processing and presentation of exogenous peptide antigen via MHC class I | 2.46E-07 | 56 | 21 |
| telomere maintenance via semi-conservative replication | 2.66E-07 | 17 | 13 |
| protein maturation | 3.21E-07 | 104 | 24 |
| antigen processing and presentation of exogenous peptide antigen via MHC class I, TAP-dependent | 3.80E-07 | 53 | 21 |
| sulfur compound metabolic process | 7.47E-07 | 130 | 22 |
| mRNA splicing, via spliceosome | 8.39E-07 | 133 | 23 |
| cytoskeleton organization | 1.09E-06 | 303 | 24 |
| cellular component movement | 1.27E-06 | 60 | 24 |
| regulation of transcription from RNA polymerase II promoter in response to hypoxia | 1.78E-06 | 21 | 19 |
| translational elongation | 2.05E-06 | 82 | 22 |
| cellular amino acid metabolic process | 2.10E-06 | 196 | 23 |
| antigen processing and presentation of exogenous peptide antigen via MHC class II | 2.19E-06 | 63 | 21 |
| transforming growth factor beta receptor signaling pathway | 2.50E-06 | 102 | 23 |
| regulation of defense response to virus by virus | 2.79E-06 | 19 | 18 |
| nucleotide-binding oligomerization domain containing signaling pathway | 3.91E-06 | 17 | 19 |
| sulfur amino acid metabolic process | 5.50E-06 | 19 | 17 |
| transcription from RNA polymerase II promoter | 9.16E-06 | 296 | 23 |
| 'de novo' posttranslational protein folding | 9.85E-06 | 26 | 18 |
| in utero embryonic development | 1.18E-05 | 180 | 24 |
| cytoskeleton-dependent intracellular transport | 1.33E-05 | 58 | 22 |
| positive regulation of type I interferon production | 1.49E-05 | 36 | 19 |
| apoptotic signaling pathway | 1.90E-05 | 68 | 21 |
| protein polyubiquitination | 2.67E-05 | 88 | 22 |
| intracellular transport of virus | 2.77E-05 | 13 | 19 |
| cell motility | 3.20E-05 | 243 | 23 |
| homeostatic process | 3.81E-05 | 343 | 24 |
| COPI coating of Golgi vesicle | 4.21E-05 | 12 | 18 |
| positive regulation of viral transcription | 4.50E-05 | 28 | 21 |
| chondroitin sulfate metabolic process | 4.66E-05 | 26 | 20 |
| keratan sulfate metabolic process | 5.44E-05 | 19 | 16 |
| ncRNA metabolic process | 5.54E-05 | 16 | 15 |
| transcription-coupled nucleotide-excision repair | 6.51E-05 | 32 | 19 |
| long-chain fatty-acyl-CoA biosynthetic process | 6.55E-05 | 13 | 15 |
| translational initiation | 7.53E-05 | 116 | 22 |
| nuclear-transcribed mRNA poly(A) tail shortening | 7.96E-05 | 25 | 17 |
| dolichol-linked oligosaccharide biosynthetic process | 9.89E-05 | 26 | 19 |
| inositol phosphate metabolic process | 9.89E-05 | 26 | 19 |
| endoplasmic reticulum unfolded protein response | 0.000105825 | 68 | 21 |
| triglyceride biosynthetic process | 0.000107744 | 31 | 21 |
| telomere maintenance via recombination | 0.000139683 | 19 | 14 |
| glucose transport | 0.000139683 | 43 | 20 |
| carbohydrate metabolic process | 0.000139683 | 426 | 24 |
| C-terminal protein lipidation | 0.000254148 | 18 | 13 |
| adherens junction organization | 0.000270642 | 27 | 21 |
| mRNA export from nucleus | 0.000400954 | 49 | 20 |
| keratan sulfate biosynthetic process | 0.000513317 | 16 | 16 |
| nucleobase-containing small molecule interconversion | 0.000522761 | 12 | 15 |
| cell proliferation | 0.000594454 | 286 | 24 |
| O-glycan processing | 0.000612419 | 26 | 17 |
| CENP-A containing nucleosome assembly | 0.000628201 | 24 | 18 |
| androgen receptor signaling pathway | 0.00063823 | 35 | 20 |
| regulation of nitric-oxide synthase activity | 0.000811823 | 16 | 16 |
| transport | 0.000866683 | 1686 | 24 |
| vacuolar transport | 0.001317206 | 41 | 20 |
| cell cycle arrest | 0.001377848 | 92 | 21 |
| unsaturated fatty acid metabolic process | 0.001558942 | 10 | 13 |
| alpha-linolenic acid metabolic process | 0.001558942 | 10 | 13 |
| negative regulation of type I interferon production | 0.001558942 | 21 | 17 |
| glucose metabolic process | 0.001576901 | 76 | 22 |
| anatomical structure morphogenesis | 0.001582612 | 50 | 20 |
| regulation of small GTPase mediated signal transduction | 0.001726041 | 96 | 22 |
| preassembly of GPI anchor in ER membrane | 0.00178448 | 11 | 9 |
| lipid metabolic process | 0.001795897 | 511 | 24 |
| regulation of transcription involved in G1/S transition of mitotic cell cycle | 0.002180006 | 19 | 18 |
| nitric oxide metabolic process | 0.002586929 | 14 | 16 |
| negative regulation of epidermal growth factor receptor signaling pathway | 0.002724591 | 29 | 17 |
| axon guidance | 0.002724591 | 196 | 24 |
| endosomal transport | 0.002838795 | 46 | 20 |
| cytokine-mediated signaling pathway | 0.003244342 | 135 | 23 |
| Ras protein signal transduction | 0.005152916 | 54 | 21 |
| RNA splicing, via transesterification reactions | 0.007502869 | 19 | 16 |
| sphingolipid biosynthetic process | 0.007623471 | 24 | 15 |
| JAK-STAT cascade involved in growth hormone signaling pathway | 0.00891557 | 14 | 15 |
| response to unfolded protein | 0.009733228 | 40 | 21 |
| skeletal system development | 0.011562927 | 97 | 23 |
| antigen processing and presentation of peptide antigen via MHC class I | 0.012460913 | 69 | 21 |
| purine nucleobase metabolic process | 0.012466241 | 22 | 19 |
| regulation of interferon-gamma-mediated signaling pathway | 0.018992916 | 14 | 14 |
| histone H4-K20 demethylation | 0.023788882 | 11 | 13 |
| retrograde vesicle-mediated transport, Golgi to ER | 0.025266241 | 25 | 20 |
| nucleotide-excision repair, DNA gap filling | 0.02642744 | 15 | 13 |
| cholesterol biosynthetic process | 0.029092539 | 28 | 19 |
| regulation of rhodopsin mediated signaling pathway | 0.033743216 | 14 | 14 |
| viral entry into host cell | 0.034830657 | 15 | 15 |
| histone mRNA metabolic process | 0.035556068 | 8 | 10 |
| response to virus | 0.039843319 | 84 | 23 |
| apoptotic process | 0.043372821 | 391 | 24 |
| plasma membrane organization | 0.043531382 | 57 | 20 |
| viral protein processing | 0.047091899 | 9 | 12 |

**Supplementary Table 5: GO categories that were enriched among predicted (MicroT-CDS) targets of miRNA that were differentially secreted by all 4 HNSCC cell lines relative to primary non-pathologic oral epithelial control cells**

| **GO Category** | **p-value** | **# miRNA Targets** | **# Associated miRNA** |
| --- | --- | --- | --- |
| cellular nitrogen compound metabolic process | 4.51E-132 | 1923 | 29 |
| biosynthetic process | 2.98E-100 | 1643 | 29 |
| cellular protein modification process | 5.75E-80 | 1017 | 29 |
| neurotrophin TRK receptor signaling pathway | 3.37E-52 | 156 | 29 |
| gene expression | 1.99E-41 | 260 | 27 |
| Fc-epsilon receptor signaling pathway | 6.90E-38 | 100 | 27 |
| small molecule metabolic process | 6.52E-33 | 855 | 28 |
| symbiosis, encompassing mutualism through parasitism | 6.52E-27 | 220 | 26 |
| viral process | 2.63E-26 | 197 | 26 |
| blood coagulation | 7.89E-25 | 199 | 29 |
| catabolic process | 1.71E-24 | 706 | 27 |
| epidermal growth factor receptor signaling pathway | 2.46E-23 | 116 | 28 |
| cellular component assembly | 1.97E-22 | 497 | 28 |
| post-translational protein modification | 9.59E-21 | 86 | 23 |
| membrane organization | 1.01E-19 | 241 | 26 |
| biological_process | 1.86E-19 | 5795 | 29 |
| synaptic transmission | 2.07E-19 | 191 | 25 |
| cell death | 6.70E-19 | 365 | 28 |
| cellular lipid metabolic process | 6.40E-18 | 77 | 25 |
| cellular protein metabolic process | 3.16E-16 | 171 | 25 |
| Fc-gamma receptor signaling pathway involved in phagocytosis | 3.95E-16 | 44 | 20 |
| cell-cell signaling | 2.89E-15 | 266 | 26 |
| mitotic cell cycle | 6.60E-15 | 149 | 26 |
| response to stress | 1.23E-14 | 782 | 29 |
| fibroblast growth factor receptor signaling pathway | 2.08E-13 | 95 | 27 |
| macromolecular complex assembly | 2.54E-13 | 320 | 27 |
| transcription, DNA-templated | 6.48E-13 | 927 | 29 |
| platelet activation | 7.76E-12 | 89 | 25 |
| glycosaminoglycan metabolic process | 2.98E-11 | 53 | 21 |
| toll-like receptor 10 signaling pathway | 5.30E-11 | 34 | 23 |
| toll-like receptor TLR1:TLR2 signaling pathway | 1.09E-10 | 35 | 23 |
| toll-like receptor TLR6:TLR2 signaling pathway | 1.09E-10 | 35 | 23 |
| nucleobase-containing compound catabolic process | 2.42E-10 | 309 | 26 |
| immune system process | 3.49E-10 | 553 | 29 |
| TRIF-dependent toll-like receptor signaling pathway | 5.01E-10 | 36 | 23 |
| transcription initiation from RNA polymerase II promoter | 6.43E-10 | 100 | 26 |
| phosphatidylinositol-mediated signaling | 1.05E-09 | 67 | 28 |
| energy reserve metabolic process | 1.34E-09 | 50 | 24 |
| toll-like receptor 5 signaling pathway | 5.26E-09 | 34 | 23 |
| toll-like receptor 9 signaling pathway | 5.66E-09 | 36 | 23 |
| cell motility | 6.57E-09 | 218 | 28 |
| axon guidance | 7.95E-09 | 189 | 25 |
| MyD88-independent toll-like receptor signaling pathway | 2.44E-08 | 36 | 23 |
| protein complex assembly | 2.95E-08 | 267 | 27 |
| cellular component disassembly involved in execution phase of apoptosis | 8.39E-08 | 25 | 16 |
| leukocyte migration | 1.70E-07 | 54 | 22 |
| protein N-linked glycosylation via asparagine | 2.07E-07 | 46 | 19 |
| cell junction assembly | 2.18E-07 | 32 | 20 |
| intrinsic apoptotic signaling pathway | 3.59E-07 | 38 | 22 |
| toll-like receptor 3 signaling pathway | 3.94E-07 | 37 | 23 |
| positive regulation of protein insertion into mitochondrial membrane involved in apoptotic signaling pathway | 4.12E-07 | 20 | 18 |
| insulin receptor signaling pathway | 4.12E-07 | 76 | 26 |
| toll-like receptor 2 signaling pathway | 4.14E-07 | 36 | 23 |
| stress-activated MAPK cascade | 6.24E-07 | 29 | 22 |
| cell junction organization | 1.27E-06 | 65 | 24 |
| extracellular matrix disassembly | 2.34E-06 | 46 | 21 |
| platelet degranulation | 3.30E-06 | 33 | 21 |
| apoptotic signaling pathway | 4.07E-06 | 60 | 22 |
| toll-like receptor 4 signaling pathway | 5.60E-06 | 41 | 24 |
| chondroitin sulfate metabolic process | 1.78E-05 | 24 | 18 |
| toll-like receptor signaling pathway | 2.51E-05 | 45 | 24 |
| extracellular matrix organization | 3.12E-05 | 137 | 26 |
| innate immune response | 3.42E-05 | 245 | 29 |
| nervous system development | 6.34E-05 | 172 | 25 |
| sulfur compound metabolic process | 9.17E-05 | 101 | 23 |
| mRNA metabolic process | 0.000104807 | 68 | 24 |
| cellular component movement | 0.000107857 | 47 | 22 |
| regulation of small GTPase mediated signal transduction | 0.000122038 | 85 | 23 |
| activation of phospholipase C activity | 0.000226757 | 30 | 20 |
| vesicle-mediated transport | 0.000234901 | 351 | 27 |
| regulation of transcription from RNA polymerase II promoter in response to hypoxia | 0.00024028 | 17 | 17 |
| hexose transport | 0.000498762 | 18 | 17 |
| vitamin metabolic process | 0.00051934 | 29 | 18 |
| generation of precursor metabolites and energy | 0.000734244 | 111 | 26 |
| water-soluble vitamin metabolic process | 0.001120686 | 26 | 18 |
| post-Golgi vesicle-mediated transport | 0.001170594 | 23 | 16 |
| DNA metabolic process | 0.00117458 | 245 | 26 |
| phospholipid metabolic process | 0.001563748 | 61 | 24 |
| MyD88-dependent toll-like receptor signaling pathway | 0.001603801 | 36 | 23 |
| cytoskeleton organization | 0.001657462 | 227 | 27 |
| glycerophospholipid biosynthetic process | 0.002217743 | 32 | 21 |
| nucleobase-containing small molecule metabolic process | 0.002423575 | 25 | 16 |
| G2/M transition of mitotic cell cycle | 0.002504931 | 54 | 24 |
| anatomical structure morphogenesis | 0.002895336 | 42 | 24 |
| regulation of glucose transport | 0.002948332 | 15 | 16 |
| RNA metabolic process | 0.002948332 | 74 | 24 |
| glutamate secretion | 0.004089972 | 15 | 18 |
| nucleotide-binding domain, leucine rich repeat containing receptor signaling pathway | 0.00441659 | 17 | 17 |
| regulation of insulin secretion | 0.005070395 | 44 | 22 |
| mitotic nuclear envelope disassembly | 0.005504013 | 15 | 17 |
| O-glycan processing | 0.005939709 | 21 | 17 |
| nuclear-transcribed mRNA catabolic process, deadenylation-dependent decay | 0.005939709 | 25 | 20 |
| G1/S transition of mitotic cell cycle | 0.005939709 | 67 | 26 |
| JAK-STAT cascade involved in growth hormone signaling pathway | 0.006107732 | 13 | 16 |
| protein polyubiquitination | 0.007083076 | 65 | 24 |
| chromatin organization | 0.007150616 | 46 | 24 |
| phosphatidylinositol biosynthetic process | 0.00803063 | 26 | 16 |
| regulation of defense response to virus by virus | 0.010314573 | 15 | 10 |
| inositol phosphate metabolic process | 0.010358466 | 20 | 15 |
| dolichol-linked oligosaccharide biosynthetic process | 0.015033579 | 17 | 14 |
| termination of RNA polymerase II transcription | 0.015424214 | 18 | 15 |
| transforming growth factor beta receptor signaling pathway | 0.015424214 | 71 | 27 |
| homeostatic process | 0.015424214 | 257 | 27 |
| nucleotide-binding oligomerization domain containing signaling pathway | 0.018714641 | 11 | 13 |
| adherens junction organization | 0.018808243 | 20 | 19 |
| in utero embryonic development | 0.029571087 | 129 | 25 |
| unsaturated fatty acid metabolic process | 0.030837134 | 7 | 8 |
| alpha-linolenic acid metabolic process | 0.030837134 | 7 | 8 |
| regulation of ubiquitin-protein ligase activity involved in mitotic cell cycle | 0.030837134 | 24 | 15 |
| transcription from RNA polymerase II promoter | 0.030837134 | 215 | 27 |
| muscle filament sliding | 0.033101848 | 16 | 15 |
| keratan sulfate metabolic process | 0.043192066 | 15 | 14 |
| regulation of cellular amino acid metabolic process | 0.046941658 | 19 | 14 |
